# The personalized approach to rituximab treatment in membranous nephropathy: a multi-center randomized controlled trial

**DOI:** 10.1016/j.eclinm.2025.103648

**Published:** 2025-11-14

**Authors:** Vesna Brglez, Maxime Teisseyre, Kévin Zorzi, Céline Fernandez, Marion Cremoni, Thomas Crepin, Antoine Lanot, Victor Gueutin, Etienne Novel-Catin, Claire Rigothier, Caroline Pelletier, Bertrand Knebelmann, Dominique Chauveau, Vincent Audard, Jean-Michel Halimi, David Verhelst, Stéphane Cambiaggio, Kevin Legueult, Laurent Bailly, Gérard Lambeau, Vincent Esnault, Olivier Moranne, Barbara Seitz-Polski

**Affiliations:** a Centre Hospitalier Universitaire de Nice, Centre de Référence Maladies Rares Syndrome Néphrotique Idiopathique, Nice, France; b Laboratoire d’Immunologie, Centre Hospitalier Universitaire de Nice, Nice, France; c Université Côte d’Azur, CNRS, INSERM, IRCAN, Nice, France; d Département de Néphrologie-Dialyse-Transplantation, Centre Hospitalier Universitaire de Nice, France; e Service de Néphrologie, Dialyse et Transplantation, Centre Hospitalier Universitaire de Besançon, Besançon, France; f Université de Franche-Comté, CHU Besançon, EFS, INSERM, UMR RIGHT, Besançon, France; g Normandie Univ, Unicaen, Centre Hospitalier Universitaire de Caen Normandie, Néphrologie, Caen, France; h Normandie Université, Unicaen, UFR de médecine, Caen, France; i ANTICIPE’’ U1086 INSERM-UCN, Centre François Baclesse, Caen, France; j Service de Néphrologie-Dialyse-Transplantation, Centre Hospitalier Universitaire de Côte de Nacre, Caen, France; k Service de Néphrologie-Dialyse, Hôpital Jacques-Monod, Flers, France; l Service de Néphrologie, Centre Hospitalier Lyon Sud, Pierre Bénite, France; m Service de Néphrologie Transplantation, Dialyse et Aphérèse, Centre Hospitalier Universitaire de Bordeaux, Bordeaux, France; n Service de Néphrologie - HTA - Dialyse, Edouard Herriot Hospital, Hospices Civils de Lyon, Lyon, France; o Service de Néphrologie-Dialyse Adultes, Assistance Publique-Hôpitaux de Paris, Université Paris Cité, Hôpital Necker Enfants Malades, Paris, France; p Département de Néphrologie et Transplantation d'organes, Centre de Référence des Maladies Rénales Rares, Centre Hospitalier Universitaire de Toulouse, INSERM U1297, Toulouse, France; q Service de Néphrologie et Transplantation, Centre de Référence Maladie Rare « Syndrome Néphrotique Idiopathique », Hôpitaux Universitaires Henri-Mondor, Assistance Publique-Hôpitaux de Paris, Créteil, France; r Univ Paris Est Créteil, Inserm U955, Institut Mondor de Recherche Biomédicale, Créteil, France; s Service de Néphrologie, Centre Hospitalier Universitaire de Tours, Tours, France; t UMR 1327, INSERM, Tours University, Tours, France; u Service de Néphrologie, Centre Hospitalier d'Avignon, Avignon, France; v Délégation de la Recherche Clinique et de l’Innovation, Université Côte d'Azur, Centre Hospitalier Universitaire de Nice, Nice, France; w Département de Santé Publique, UR2CA, Université Côte d'Azur, Centre Hospitalier Universitaire de Nice, Nice, France; x Université Côte d'Azur - Centre Hospitalier Universitaire de Nice, UR2CA, Nice, France; y Centre National de la Recherche Scientifique, Inserm, Institut de Pharmacologie Moléculaire et Cellulaire, Sophia Antipolis, Université Côte d'Azur (UniCa), Valbonne Sophia Antipolis, France; z Service de Néphrologie Dialyse Aphérèse, Centre Hospitalier Universitaire de Nîmes, Nîmes, France; aa IDESP Université de Montpellier, Montpellier, France

**Keywords:** Membranous nephropathy, Personalized treatment, Rituximab, Epitope spreading, Clinical trial

## Abstract

**Background:**

Membranous nephropathy is a renal autoimmune disease associated with autoantibodies against phospholipase A2 receptor (PLA2R1) in 50–80% of cases. Patients develop immunity towards a single or multiple PLA2R1 domains, defining a cascade immunization or epitope spreading associated with worse prognosis and low rate of spontaneous remission. We aimed to compare the efficacy of standard versus personalized treatment (based on biomarker: epitope spreading) with the immunosuppressor rituximab on remission rate at month-12.

**Methods:**

A randomized, prospective clinical trial (NCT03804359) was conducted in 12 French hospitals. Enrollment between November 2019 and October 2022. Follow-up lasted two years after inclusion and ended in October 2024. Sixty-four patients with PLA2R1-associated membranous nephropathy were randomly assigned (1:1) to the GEMRITUX protocol (symptomatic treatment for six months followed by two 375 mg/m^2^ rituximab infusions at month-6 in case of persistent nephrotic syndrome) or to the personalized protocol (patients without epitope spreading at month-0/month-6 followed the GEMRITUX protocol, while patients with epitope spreading at month-0/month-6 were treated immediately with two 1 g rituximab infusions). The primary outcome was the combined endpoint of partial or complete clinical remission at month-12.

**Findings:**

Thirty-one patients (48%) were randomized to the GEMRITUX arm and 33 (52%) to the personalized arm. In the GEMRITUX arm, four patients were treated with NIAT only since they entered into spontaneous remission at month-6, while 24 patients received low dose rituximab at month-6. In the personalized arm stratified according to their epitope spreading status, non-spreaders (n = 16) received NIAT for six months, while spreaders (n = 17) were immediately treated with high-dose rituximab. At month-12, the clinical remission rate was higher in the personalized group (67% versus 35%, p = 0.01), associated with improved kidney function (p = 0.0498 for estimated glomerular filtration rate). There was no difference in the rate of spontaneous remission nor in the number of adverse events between both groups suggesting that patients in the personalized arm were not over-treated.

**Interpretation:**

Personalized treatment protocol based on PLA2R1 epitope spreading status is superior to the standard GEMRITUX protocol in achieving clinical remission at month-12 by stratifying the patients according to the immunological severity of the disease and by immediately treating the patients at risk of treatment failure with rituximab.

**Funding:**

10.13039/501100009243DGOS, PHRC National 2017.

**Research in context**

**Evidence before this study**

Optimal treatment in patients with membranous nephropathy, a rare autoimmune kidney disease, is crucial in preventing loss of kidney and maintaining patients’ quality of life. However, since immunosuppressive treatment can have important side effects, it is important to carefully select the patients who really need the treatment. We searched PubMed database, from database inception to September 30th, 2025, for papers published in English, using the terms “membranous nephropathy clinical trial”, “membranous nephropathy epitope”, and “membranous nephropathy rituximab”. Our search yielded 341, 140 and 554 results, respectively.

**Added value of this study**

Improved stratification and treatment protocol described in this study, based on a blood immune biomarker, helps select the patients in need of an immunosuppressive treatment while not treating the patients that might recover without treatment. In comparison to the standard treatment regimen, the personalized protocol significantly improves clinical remission rates and maintains a better kidney function.

**Implications of all the available evidence**

These findings can help optimize the treatment regimen in membranous nephropathy patients by carefully selecting the patients in need of immediate immunosuppressive treatment, without compromising their kidney function or exposing them to unnecessary side effects.

## Introduction

Membranous nephropathy (MN) is a rare, but severe autoimmune disease.^1^^,^^2^ Clinical evolution is variable, ranging from spontaneous remission in about one third of patients, persistent nephrotic syndrome in another third, and kidney failure in the last third.^1^^,^^2^ Among more than 10 autoantigens implicated in MN,^3^ the M-type phospholipase A2 receptor (PLA2R1) is present in 50–80% of MN patients.^4^, ^5^, ^6^, ^7^, ^8^ The titer of anti-PLA2R1 antibodies reflects the clinical status of the patient, i.e. high anti-PLA2R1 titer at diagnosis and during follow-up is associated with a worse clinical condition and a poor clinical outcome.^9^^,^^10^ Since the rise and fall of anti-PLA2R1 titer precede the deterioration and improvement, respectively, of clinical markers of kidney function, it has become an invaluable tool both for the diagnosis and monitoring of MN patients.^11^^,^^12^

Kidney Disease Improving Global Outcomes (KDIGO) guidelines recommend a supportive symptomatic treatment with blockers of the renin-angiotensin system and diuretics, and immunosuppressive therapy only in the case of renal function deterioration, complication or persistent nephrotic syndrome according to the risk of progression (based on estimated glomerular filtration rate (eGFR), hypoalbuminemia, anti-PLA2R1 antibody titer or proteinuria).^13^ Rituximab, an anti-CD20 antibody, is a relatively safe and efficient treatment option in MN patients as demonstrated in several clinical trials.^14^, ^15^, ^16^ KDIGO guidelines now recommend rituximab as a first line treatment.^13^

In addition to the quantity of anti-PLA2R1 antibodies, their epitope specificity may also be important.^17^ Patients may present antibodies recognizing only the cysteine-rich domain (CysR) of PLA2R1, or may additionally recognize C-type lectin domains (CTLD) 1, 7 and 8^18^, ^19^, ^20^ via a mechanism of intramolecular epitope spreading associated with poor clinical presentation at baseline and worse prognosis.^21^, ^22^, ^23^ Epitope spreading is a well described phenomenon in many autoimmune diseases^24^, ^25^, ^26^; including in Heymann nephritis, a rat model of MN.^27^ Epitope spreading was an independent factor of remission in the randomized controlled trial GEMRITUX comparing low-dose rituximab (two 375 mg/m^2^ injections) versus placebo.^22^ In response to symptomatic treatment, after 6 months, spreaders had 0.05% chance of entering into spontaneous remission with symptomatic treatment alone, versus 45% for non-spreaders. Accordingly, non-spreader patients entered into rituximab-induced remission regardless of the rituximab dose used, while spreader patients had higher chances of achieving remission with high dose rituximab (two injections of 1 g) versus low dose (two injections of 375 mg/m^2^).^28^ Clinical significance of epitope spreading nevertheless remains a matter of lively debate.^20^^,^^29^^,^^30^

We designed this randomized controlled study to investigate whether a personalized treatment approach based on the rituximab regimen tailored to the epitope spreading status of the patient (marker-based strategy design)^31^ is superior to the standard GEMRITUX protocol in achieving remission at month-12 for patients with PLA2R1-associated membranous nephropathy.

## Methods

### Study design and oversight

This is a two-armed, randomized, open-label, multicenter, prospective trial using the marker-based strategy design integrating the biomarker in the treatment strategy, as described previously.^31^ In more detail, the study scheme corresponds to Figure 2A from Sargent et al.^31^ Test marker is the determination of epitope spreading. Treatment A is the standard GEMRITUX treatment, while treatment B is the personalized treatment based on the epitope spreading status.

The study was conducted in 12 French hospitals. The list of study investigators for each center and coordination committee members is available in Supplementary data. Clinical trial was registered under NCT03804359 (Personalized Medicine for Membranous Nephropathy PMMN) on 11th January 2019. Trial design was approved by the local ethics committee Sud-Ouest et Outre mer II (approval 2-19-035-id3464). Detailed study protocol has already been published.^32^

The study lasted five years: inclusion period lasted three years with two years of follow-up. Follow-up visits were scheduled at month-3, month-6, month-9, month-12, month-18 and month-24. At each visit, a complete clinical and immunological work-up was performed, including weight, systolic and diastolic blood pressure, serum creatinine, serum albumin, eGFR, UPCR, urine albumin to creatinine ratio, anti-PLA2R1 titer, PLA2R1 epitope spreading status, residual serum rituximab level, titer of anti-rituximab antibodies, presence of edema, type of concomitant and MN-related treatment received, adverse events.

### Ethics statement

All patients signed a written informed consent for the participation in the study and the study adhered to the declaration of Helsinki.

### Inclusion and exclusion criteria

Inclusion criteria were: aged 18 years or more, anti-PLA2R1 activity detected by enzyme-linked immunosorbent assay (ELISA) or immunofluorescence (IF) assay, nephrotic syndrome defined by proteinuria >3.5 g/24 h (or urine protein to creatinine ratio (UPCR) > 3.5 g/g) and serum albumin <30 g/L, eGFR (CKD-EPI formula) > 30 mL/min/1.73 m^2^, symptomatic treatment according to KDIGO guidelines: maximal tolerated dose of non-immunosuppressive antiproteinuric treatment NIAT (angiotensin-converting enzyme inhibitor and/or angiotensin 2 receptor blockers), medical insurance, provided a signed informed consent, having understood and accepted the need for long-term medical follow-up, and an effective mode of contraception for women in child-bearing age.

Exclusion criteria were: secondary MN (MN related to cancers, infections, systemic lupus erythematosus, drugs), anti-PLA2R1 antibodies not confirmed by central analysis, pregnancy or breastfeeding, immunosuppressive treatment in the last six months, cancer under treatment, complicated nephrotic syndrome that would require early immunosuppressive treatment (thrombosis, acute renal failure), severe infections or active hepatitis B, hypersensitivity to the active substance or to murine proteins or to any of the other excipients, severely immunocompromised state, severe heart failure (New York Heart Association Class IV) or severe uncontrolled cardiac disease, presence of anti-rituximab antibodies in relapsing MN patients, and unable to give an informed consent.

Block randomization was centralized and balanced (1:1) using REDCap.^33^ The allocation to each arm was entirely random and was not stratified by the participating centers nor by any of the study variables, such as epitope spreading status, proteinuria, BMI, eGFR etc. Since this is an open-label study and since the study design required the investigators to adapt the treatment according to the epitope spreading status of their patients, the investigators were not blinded to the treatment received nor to the epitope spreading status of the patient.

### Sample size calculation

#### Estimated remission rate in the personalized arm

According to our preliminary data, about 30% of patients with MN have CysR-restricted activity at diagnosis.^21^ About 50% will enter in spontaneous remission after six months of NIAT and 40% after NIAT + Rituximab at low dose (375 mg/m^2^ D0 and D7).^22^ About 70% of patients have CTLD1 and/or CTLD7 activity in our cohorts, and about 85% entered in remission after repeated pulses of high doses of rituximab.^28^ We therefore expect a remission rate of 80% at M12 in the personalized arm.

#### Estimated remission rate in GEMRITUX arm

Based on data from the literature, 10–21% of MN patient with nephrotic syndrome entered into spontaneous remission after 1-year of NIAT^1^^,^^14^ and 35% in GEMRITUX after NIAT + low doses of rituximab (375 mg/m^2^ D0 and D7).^14^ We expect a remission rate of 45% in the control arm.

#### Sample size

With α = 0.05 and β = 0.20 (two-sided test), the number of patients required is 29 in each group (Nquery Advisor v 7.0, two group Fisher's-exact test). To account for a 10% rate of loss to follow-up (anticipated as minor in this study as patients are intensively followed for this pathology), the global sample size is 64 patients.

With an average of one or two patients eligible for the study in each participating center yearly, and since 50% of patients may refuse to participate, we estimated that four years were required to recruit the calculated sample size. Finally, the 64 patients were included in only three years.

### Treatment and follow-up

#### GEMRITUX arm

Patients from the GEMRITUX group were treated as per GEMRITUX protocol,^14^ i.e. they received NIAT for six months after inclusion. At month-6 either the patient entered into remission and continued receiving NIAT only, or the patient remained nephrotic (UPCR >3.5 g/g) and was treated with two rituximab infusions at 375 mg/m^2^ at 1-week interval.

#### Personalized arm

Patients in the personalized group were stratified at inclusion according to their PLA2R1 epitope profile into non-spreaders (antibodies against CysR domain only) and spreaders (antibodies against CysR domain and CTLD1 and/or CTLD7 domains).

Non-spreaders were treated with NIAT only for six months. At month-6 their treatment was modified accordingly: a) if the patient was in remission (partial or complete), NIAT was continued; b) if the epitope profile remained unchanged with CysR-restricted activity and active disease (UPCR >3.5 g/g), the patient was treated with low dose rituximab (i.e. two 375 mg/m^2^ rituximab infusions at 1-week interval). c) If the patient developed additional antibodies against CTLD1 and/or CTLD7 domains at month-6 and had active disease (UPCR >3.5 g/g), they were treated with high dose rituximab (i.e. two 1 g rituximab infusions at 2-week interval).

Patients with epitope spreading at month-0 were treated with high dose rituximab (i.e. two 1 g rituximab infusions at 2-week interval) immediately after inclusion. Patients were reevaluated at month-6 and the treatment adapted accordingly: a) NIAT only for patients in remission (partial or complete); b) a second therapeutic intervention with low dose rituximab (i.e. two 375 mg/m^2^ rituximab infusions at 1-week interval) for patients with CysR-restricted activity and active disease (UPCR >3.5 g/g); c) a second therapeutic intervention with high dose rituximab (i.e. two 1 g rituximab infusions at 2-week interval) for spreader patients with active disease (UPCR >3.5 g/g).

After month-12, the participating centers were free to choose any treatment they deemed appropriate.

### Study endpoints

Primary outcome was a combined endpoint of partial (UPCR <3.5 g/g with a decrease greater than 50% from baseline, serum albumin >30 g/L, increase of serum creatinine lower than 20% at month-12) or complete (UPCR <0.3 g/g, serum albumin >35 g/L, eGFR >60 mL/min/1.73 m^2^) clinical remission at month-12.

Secondary endpoints were: a) immunological remission (i.e. anti-PLA2R1 titer <14 RU/mL); b) complete clinical remission; c) evolution of albumin, creatinine, eGFR, UPCR, and anti-PLA2R1 between month-0 and month-12; d) occurrence of serious adverse events; e) residual serum rituximab level three months after the first injection; f) occurrence of anti-rituximab antibodies.

Exploratory analyses not initially planned in the study protocol were: a) subgroup analyses on patients stratified by their epitope spreading status, anti-PLA2R1 titer, BMI, cumulative rituximab dose received and history of MN prior to inclusion in the study (rituximab as first line treatment or relapsing patients); b) multivariate analyses of factors influencing remission at month-12, rituximab treatment regimen factors influencing remission and eGFR at month-12; c) rate of spontaneous remission at month-12; d) time to remission; e) remission rate six months after first rituximab injection.

### Laboratory measurements

Anti-PLA2R1 titer and PLA2R1 epitope profile were determined with an ELISA from Euroimmun (Germany) and home-made ELISA, respectively, as described previously.^21^^,^^22^^,^^32^ Residual rituximab level and anti-rituximab antibodies were quantified by ELISA (LISA-TRACKER, Theradiag, France).

### Statistical analyses

Final analyses of the primary endpoint at month-12 and secondary endpoints until month-12 were performed after the database lock when all included patients completed the month-12 visit. Primary endpoint was analyzed by the intention to treat principle using worst case scenario to impute missing data to minimize bias (i.e. no remission for the patients lost to follow-up from the personalized arm, and remission for the patients lost to follow-up from the GEMRITUX arm). Secondary endpoints were analyzed using the last observation carried forward (LOCF) principle.

Quantitative variables are presented as mean and standard deviation or median and interquartile range depending on whether the data had a Gaussian distribution or not. Qualitative variables are presented as numbers and percentages. D'Agostino & Pearson test was used to assess the normality of the variables (GraphPad Software, Inc., San Diego, CA, USA). Continuous data were compared using the Student t-test or Mann–Whitney test as appropriate. Qualitative variables were compared using Pearson Chi-squared test or Fisher's exact test in case of small samples. Kaplan–Meier and log-rank test were used to assess the remission rate at different time points. Multivariate logistic regression models were used to investigate independent predictive factors of clinical remission depending on the treatment group, adjusted for the potential confounding factors. All comparisons were two-tailed, and p-values <0.05 were considered as significant.

Statistical analyses were performed using GraphPad Prism 8 (GraphPad Software, Inc., San Diego, CA, USA) and SAS Enterprise Guide 7.15 (Cary, NC, USA).

### Role of the funding source

DGOS (Direction Générale de l'Offre de Soins) who funded this clinical trial via PHRC National 2017 had no role in study design, data collection, data analysis, data interpretation, or writing of the report.

## Results

### Cohort description

Sixty-four patients were included between November 2019 and October 2022. Follow-up lasted two years after inclusion and ended in October 2024.

**Fig. 1:**
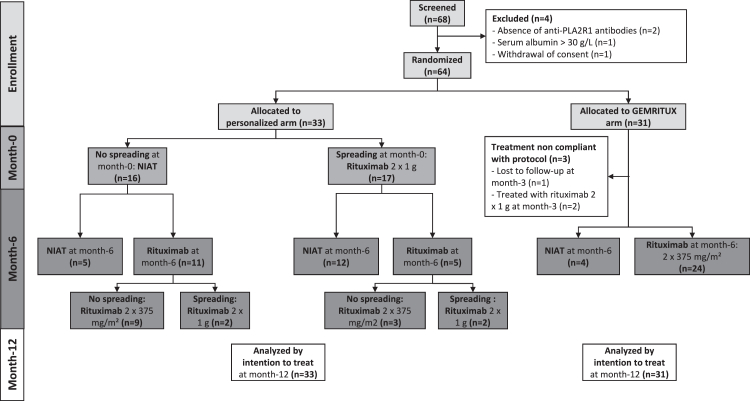
Study flowchart. From the 68 patients screened, 64 were included in the final analysis of the primary endpoint following the intention to treat principle. For two patients (3%) who were lost to follow-up before month-12, the worst-case scenario was used to impute missing data. From the 64 patients included, 33 (52%) were included in the personalized arm, and 31 (48%) in the GEMRITUX arm. Patients from the GEMRITUX arm received NIAT only for six months. At month-6, either the patients achieved spontaneous remission (n = 4), in which case NIAT only was continued, or they had an active disease (n = 24), in which case they were treated with two 375 mg/m^2^ rituximab injections at one week interval. Three patients from GEMRITUX arm did not receive allocated treatment due to loss to follow-up before month-6 (n = 1) and premature treatment with a high dose rituximab (two injections of 1 g) at month-3 since their symptoms worsened (n = 2). Patients from the personalized arm were stratified at month-0 according to their epitope spreading status. Patients without epitope spreading (n = 16) received NIAT only for six months. Patients with epitope spreading (n = 17) were treated immediately with two 1 g rituximab injections at two weeks interval. The clinical and immunological profile of patients in the personalized arm was reassessed at month-6. Patients in spontaneous (n = 5) or rituximab-induced remission (n = 12) received NIAT only. Patients with active disease and without epitope spreading at month-6 were treated (n = 9 for patients without epitope spreading at month-0) or retreated (n = 3 for patients with epitope spreading at month-0 who have already received rituximab at month-0) with two 375 mg/m^2^ rituximab injections at one week interval. Patients with active disease and with epitope spreading at month-6 were treated (n = 2 for patients without epitope spreading at month-0) or retreated (n = 2 for patients with epitope spreading at month-0 who have already received rituximab at month-0) with two 1 g rituximab injections at two weeks interval. All 64 patients were included in the final analysis of combined endpoint of partial or complete clinical remission at month-12. NIAT: non-immunosuppressive antiproteinuric treatment; PLA2R1, phospholipase A2 receptor 1.

**Fig. 2:**
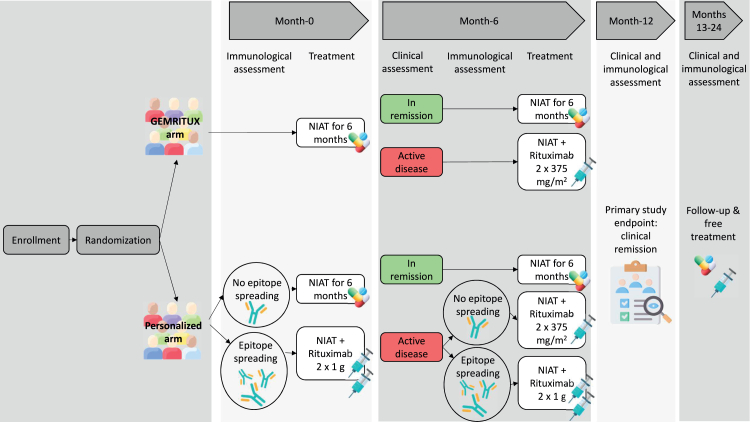
Study design. Patients included in the study were randomized at enrollment to either standard (GEMRITUX arm) or personalized treatment (personalized arm). Patients in the GEMRITUX arm were treated with NIAT only for six months. In case of spontaneous remission at month-6, NIAT was continued. In case of persistent nephrotic syndrome, patients were treated with a low dose rituximab. Patients in the personalized arm were stratified at inclusion according to their epitope spreading status. Patients without epitope spreading were treated with NIAT only for six months, while patients with epitope spreading received two injections of high dose rituximab. The clinical and immunological profile of patients in the personalized arm was reassessed at month-6. Patients in spontaneous or rituximab-induced remission at month-6 received NIAT only for the following six months. Patients with clinically active disease were treated with either low dose rituximab in case of immunologically mild disease without epitope spreading, or high dose rituximab in case of immunologically severe disease with epitope spreading. The primary outcome (combined endpoint of partial or complete clinical remission) was evaluated at month-12. Patients were continuously monitored until month-24 (end of the study). The choice of treatment between month-12 and month-24 was left to each participating center.

According to the intention to treat principle, all 64 patients included were analyzed. Thirty-one patients (48%) were randomized in the GEMRITUX arm and 33 (52%) in the personalized arm. Study flowchart and study design are presented in Figs. 1 and 2, respectively. Fifty-one patients (80%) were male, the median age was 62 years, and the median anti-PLA2R1 titer was 75 RU/mL (Table 1, Supplementary Table S1). There were no differences in age, gender, blood pressure, serum albumin, UPCR, edema, anti-PLA2R1 titer, anti-PLA2R1 titer higher than the median of 75 RU/mL, rate of PLA2R1 epitope spreading, KDIGO risk classification, time since the onset of symptoms, ratio of relapsing and first course patients or the type of symptomatic treatment received between the two arms at baseline. Patients from the personalized arm had a significantly higher body mass index (BMI) than patients from the GEMRITUX arm (28.0 ± 5.0 versus 25.3 ± 4.0, respectively, p = 0.02) which was unrelated to the edema that were similar in both experimental arms (p > 0.99). Serum creatinine was significantly lower in the GEMRITUX arm than in the personalized arm (91.0 [79.0; 108.0] versus 117.0 [86.5; 143.5] μmol/L, respectively, p = 0.009), while eGFR was higher in the GEMRITUX arm than in the personalized arm (73.6 ± 21.6 versus 60.8 ± 23.7 mL/min/1.73 m^2^, respectively, p = 0.03). These differences were not related to the number of patients receiving NIAT which was similar in both experimental arms (p = 0.32, 0.51 and > 0.99 for anti-hypertensive treatment, RAAS blockers and other symptomatic treatment, respectively).

**Table 1:** Baseline characteristics.

	All (n = 64)	GEMRITUX arm (n = 31)	Personalized arm (n = 33)	p-value
Age (years)	61.8 ± 12.5	61.8 ± 13.2	61.9 ± 12.0	0.98
Men (n, %)	51 (80%)	23 (74%)	28 (85%)	0.36
Height (cm)	172 ± 8^a^	171 ± 9^a^	174 ± 8	0.52
Weight (kg)	79.4 ± 15.5^a^	74.4 ± 14.9	84.2 ± 14.8^a^	**0.01**
BMI (kg/m^2^)	26.7 ± 4.7^b^	25.3 ± 4.0^a^	28.0 ± 5.0^a^	**0.02**
Systolic BP (mmHg)	138 ± 18	136 ± 19	139 ± 16	0.51
Diastolic BP (mmHg)	83 ± 12	83 ± 13	83 ± 11	0.78
Creatinine (μmol/L)	98.5 [83.4; 126.8]	91.0 [79.0; 108.0]	117.0 [86.5; 143.5]	**0.009**
Albumin (g/L)	24.7 ± 4.0	25.1 ± 3.6	24.4 ± 4.4	0.47
eGFR (mL/min/1.73 m^2^)	67.0 ± 23.5	73.6 ± 21.6	60.8 ± 23.7	**0.03**
UPCR (g/g)	7.1 ± 2.5^a^	7.0 ± 2.7	7.1 ± 2.5^a^	0.85
Edema	31 (48%)	15 (48%)	16 (48%)	>0.99
Anti-PLA2R1 (RU/mL)	75 [28; 168]^b^	69 [25; 278]^a^	89 [29; 149]^a^	0.85
Anti-PLA2R1 > 75 RU/mL (n, %)	31 (50%)^b^	13 (43%)^a^	18 (56%)^a^	0.45
Epitope spreading (n, %)	32 (51%)^a^	15 (50%)^a^	17 (52%)^c^	>0.99
KDIGO risk classification (n, %)	Moderate risk: 8 (13%) High risk: 56 (87%)	Moderate risk: 4 (13%) High risk: 27 (87%)	Moderate risk: 4 (12%) High risk: 29 (88%)	>0.99
Time since the onset of symptoms (months)	4.0 [2.3; 7.7]	4.4 [2.5; 8.8]	3.4 [2.1; 7.6]	0.30
History of MN (n, %)	First course: 44 (69%) Relapse: 20 (31%)	First course: 19 (61%) Relapse: 12 (39%)	First course: 25 (76%) Relapse: 8 (24%)	0.28
Anti-hypertensive treatment: diuretics, beta blockers, calcium inhibitors (n, %)	None: 10 (16%) 1 drug: 31 (48%) 2 drugs: 16 (25%) 3 drugs: 7 (10%)	None: 7 (23%) 1 drug: 12 (39%) 2 drugs: 9 (29%) 3 drugs: 3 (10%)	None: 3 (9%) 1 drug: 19 (58%) 2 drugs: 7 (21%) 3 drugs: 4 (12%)	0.32
RAAS blockers (n, %)	None: 1 (2%) 1 drug: 60 (94%) 2 drugs: 3 (5%)	None: 0 (0%) 1 drug: 29 (94%) 2 drugs: 2 (6%)	None: 1 (3%) 1 drug: 31 (94%) 2 drugs: 1 (3%)	0.51
Other symptomatic treatment (n, %)	None: 59 (92%) 1 drug: 5 (8%)	None: 29 (94%) 1 drug: 2 (6%)	None: 30 (91%) 1 drug: 3 (9%)	>0.99

BP, blood pressure; eGFR, estimated glomerular filtration rate; BMI, body mass index; PLA2R1, phospholipase A2 receptor; MN, membranous nephropathy; UPCR, urine protein to creatinine ratio; RAAS, renin-angiotensin-aldosterone system.

Bolded p values indicate statistical significance (p < 0.05).

a Missing value for one patient.

b Missing values for two patients.

c Missing value for one patient in the personalized arm without an immunological workup at month-0 was replaced with a previous immunological workup at screening one month before enrollment in the study. Of note, the epitope spreading result (i.e. no spreading) was identical pre-enrollment and at month-3 follow-up visit.

### Treatment

Before month-12, 54 patients (86%) received at least one rituximab dose (Fig. 1, Table 2). In the GEMRITUX arm, four patients were treated with NIAT only since they entered into spontaneous remission at month-6, while 24 patients received low dose rituximab at month-6. Patients in the personalized arm were stratified according to their epitope spreading status at baseline. Non-spreaders (n = 16) received NIAT for six months, while spreaders (n = 17) were immediately treated with high dose rituximab. At month-6, patients in the personalized arm who were in either spontaneous (n = 5) or rituximab-induced (n = 12) remission continued receiving NIAT only. The patients with still active disease at month-6 were treated according to their epitope spreading status: low dose rituximab for non-spreaders (n = 12) and high dose rituximab for spreaders (n = 4). While there was no significant difference in the number of rituximab injections per patient between both arms (p = 0.08), the patients from the personalized arm received a higher cumulative dose of rituximab than the patients from the GEMRITUX arm (2000 [1445; 2000] versus 1350 [1200; 1500] mg, respectively, p < 0.001). The residual level of rituximab did not differ between the groups (p = 0.68). Twelve (19%) patients newly developed anti-rituximab antibodies at month-12 and there was no difference in the probability of developing anti-rituximab antibodies between groups (p = 0.55).

**Table 2:** Treatment received between month-0 and month-12.

	All (n = 64)	GEMRITUX (n = 31)	Personalized (n = 33)	p-value
Type of treatment before month-12 (n, %)	NIAT only: 8 (13%) Rituximab: 54 (86%) Obinutuzumab: 1 (2%)^a^	NIAT only: 4 (13%) Rituximab: 26 (87%) Obinutuzumab: 0 (0%)^a^	NIAT only: 4 (12%) Rituximab: 28 (85%) Obinutuzumab: 1 (3%)	0.63
Nr of rituximab injections before month-12 (n)	0: 4 2: 47 4: 5^a^	0: 4 2: 26 4: 0^a^	0: 4 2: 23 4: 5	0.08
Cumulative rituximab dose per patient before month-12 (mg)	1500 [1260; 2000]	1350 [1200; 1500]	2000 [1445; 2000]	**<0.001**
Patients with detectable serum rituximab level 3 months after first injection (n, %)	6 (12%)^b^	2 (9%)^c^	4 (15%)^d^	0.68
Patients with anti-rituximab antibodies at month-0 (n, %)	2 (3%)	0 (0%)	2 (6%)	0.49
Patients with anti-rituximab antibodies at month-12 (n, %)^e^	14 (22%)	8 (26%)	6 (18%)	0.55

NIAT, nonimmunosuppressive antiproteinuric treatment.

Bolded p values indicate statistical significance (p < 0.05).

a Missing value for one patient (lost to follow-up before treatment initiation).

b Missing values for five out of 54 patients treated with rituximab.

c Missing values for four out of 26 patients treated with rituximab.

d Missing value for one out of 28 patients treated with rituximab.

e LOCF was used to impute missing data.

### Primary outcome: partial or complete clinical remission at month-12

**Fig. 3:**
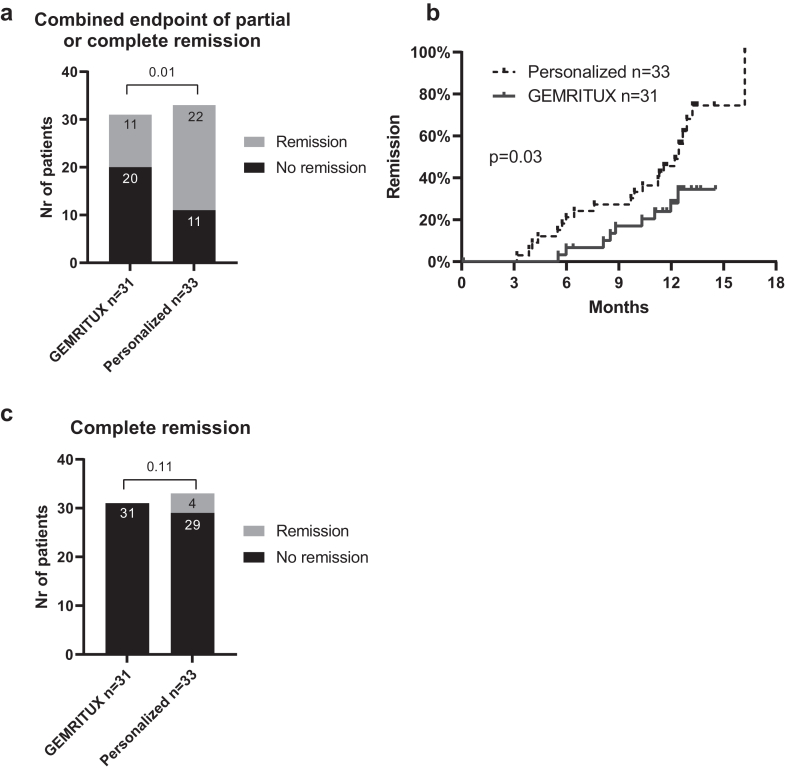
Remission at month-12. a) Primary endpoint of partial or complete remission at month-12. Eleven patients (35%) from GEMRITUX arm achieved the composite endpoint of partial or complete remission at month-12, versus 22 (67%) from the personalized arm (p = 0.01). Worst case scenario method was used to impute missing data. b) Time to remission. Patients from the personalized group achieved the primary endpoint (partial or complete clinical remission) at a faster rate than patients from the GEMRITUX arm (p = 0.03). LOCF principle was used to impute missing data. For a patient lost to follow-up immediately after inclusion in the study, the time 0.1 months was arbitrarily assigned in order to be able to display it on Kaplan–Meier plot. c) Complete remission at month-12. Four patients (6%) from the personalized arm achieved complete remission at month-12, while no patient from the GEMRITUX arm achieved complete remission (p = 0.11). LOCF principle was used to impute missing data. LOCF, last observation carried forward.

At month-12, 33 patients (52%) achieved the combined endpoint of partial or complete clinical remission (Supplementary Table S2, Fig. 3a). Patients from the personalized group had a higher chance of entering into remission than the GEMRITUX group (67% versus 35% of patients, respectively, p = 0.01).

Since the BMI was statistically different between both experimental arms, we validated the results of the primary outcome with the analysis of patients with BMI below or above 30 kg/m^2^ (Supplementary Table S3). Non-obese patients were more likely to achieve remission with personalized treatment (p = 0.009), while there was no difference in the outcome for the patients with BMI >30 kg/m^2^ (p = 0.50). A multivariate analysis (Supplementary Table S4) demonstrated that the experimental arm and BMI, but not eGFR, were independent prediction factors of the combined endpoint at month-12 (p = 0.003, p = 0.01 and p = 0.96, respectively).

**Fig. 4:**
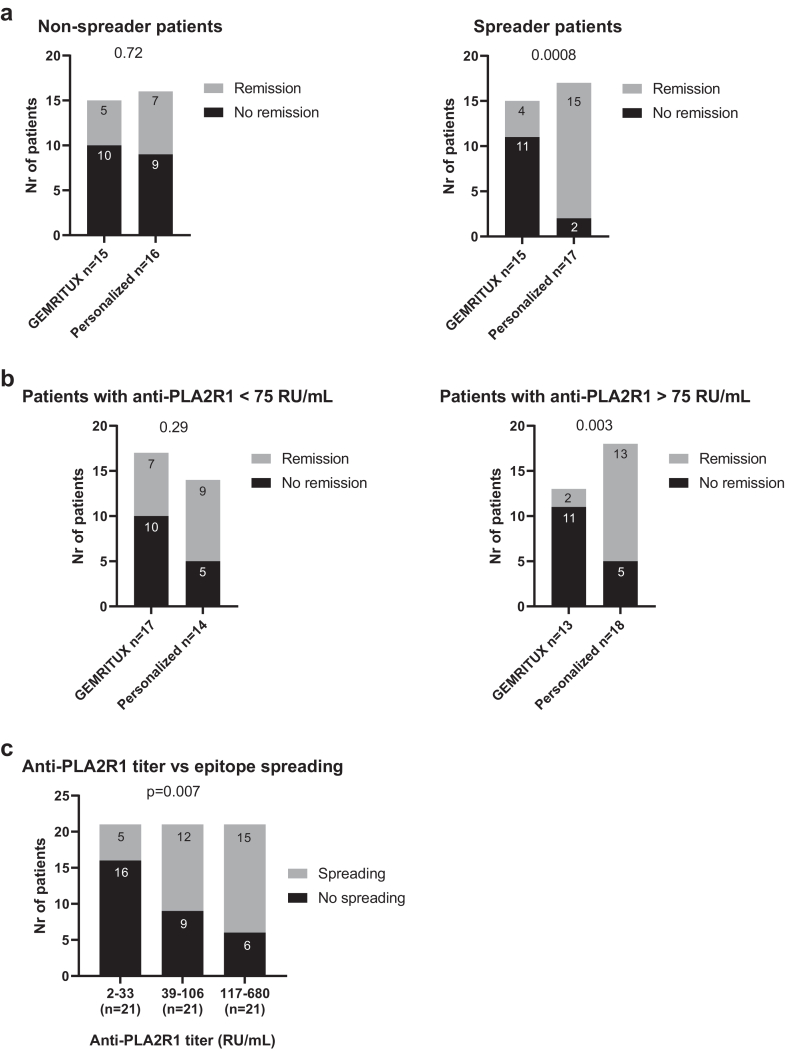
Remission at month-12 in subgroups depending on anti-PLA2R1 titer and/or epitope spreading. a) Primary endpoint of partial or complete remission at month-12 for the subgroup of patients without (left panel) and with (right panel) epitope spreading at month-0. While there was no difference in the remission rates between the two experimental arms for non-spreader patients (p = 0.72), spreader patients were more likely to achieve remission if treated with personalized protocol (p = 0.0008). Epitope spreading status at baseline was missing for one patient in the GEMRITUX arm. b) Primary endpoint of partial or complete remission at month-12 in patients with anti-PLA2R1 titer at month-0 either below (left panel) and above (right panel) the median of 75 RU/mL. While there was no difference in remission rates between the two experimental arms for the patients with anti-PLA2R1 titer lower than 75 RU/mL (p = 0.29), patients with high titer were more likely to achieve remission if treated with the personalized protocol (p = 0.003). Anti-PLA2R1 titer at baseline was missing for one patient in each arm. c) The association between anti-PLA2R1 titer and epitope spreading at baseline. The proportion of patients with epitope spreading increases as the anti-PLA2R1 titer increases as well (p = 0.007 when the patients are divided into tertiles according to their anti-PLA2R1 titer. Epitope spreading status at baseline was missing for one patient. LOCF was used to impute missing data for remission at month-12 for panels a and b, and to impute missing data for anti-PLA2R1 titer for panel c. LOCF, last observation carried forward; PLA2R1, phospholipase A2 receptor 1.

There was no difference in remission rates between both experimental arms for non-spreaders at month-0 (33% versus 44% for GEMRITUX and personalized arm, respectively, p = 0.72) (Fig. 4a). For spreader patients, 27% of patients from GEMRITUX arm and 88% from personalized arm achieved the combined endpoint (p = 0.0008). The difference in remission rates between both arms was correlated to anti-PLA2R1 titer at month-0 (patients with anti-PLA2R1 titer below median of 75 RU/mL: 41% versus 64% of remission for GEMRITUX and personalized arm, respectively, p = 0.29; patients with anti-PLA2R1 titer above median of 75 RU/mL: 15% versus 72% of remission for GEMRITUX and personalized arm, respectively, p = 0.003) (Fig. 4b). Higher anti-PLA2R1 titer was associated with a higher proportion of epitope spreading (p = 0.007) (Fig. 4c).

The difference in remission rates was correlated to KDIGO classification (no difference between both arms for patients at moderate risk according to KDIGO, while the patients at high risk according to KDIGO were more likely to enter into remission in the personalized arm than in the GEMRITUX arm, 69% versus 37%, respectively, p = 0.02) (Supplementary Figure S1A). High risk according to KDIGO was associated with a higher proportion of epitope spreading in comparison to patients with moderate risk according to KDIGO (p = 0.005) (Supplementary Figure S1B).

From the 44 newly diagnosed patients with no previous immunosuppressive treatment, five patients (26%) from the GEMRITUX arm and 17 patients (68%) from the personalized arm achieved the combined endpoint at month-12 (p = 0.01, Supplementary Figure S2). The difference in remission rates between both arms was not statistically significant for relapsing patients (33% versus 63% for GEMRITUX and personalized arm, respectively, p = 0.36), although the number of events was probably too small to conclude with certainty.

The remission rate six months after the first rituximab injection was not different between both experimental arms (p = 0.99, Supplementary Figure S3), and the remission rate at month-12 was independent of the cumulative dose of rituximab received (p = 0.32, Supplementary Figure S4), while the delay between the inclusion and first rituximab injection was significantly different between both experimental arms (p = 0.0004, Supplementary Figure S5). Multivariable analysis confirmed that the remission at month-12 was associated with the delay between inclusion and first rituximab injection, but not with cumulative rituximab dose received (p = 0.002 and p = 0.15, respectively, Supplementary Table S5).

Five patients (16%) entered into spontaneous remission at month-12, and there was no difference in the rate of spontaneous remission between both arms (p > 0.99, Supplementary Table S2). The time to remission as depicted by Kaplan–Meier curve was different between both arms (Fig. 3b, p = 0.03).

Patients in the personalized arm were more likely to achieve the combined endpoint also in the per protocol analysis conducted on 54 patients without any deviation from the protocol (36% versus 69% remission for the GEMRITUX and personalized arm, respectively, p = 0.02, Supplementary Figures S6 and S7).

### Secondary outcomes

Four patients (6%), all from the personalized arm, entered into complete clinical remission at month-12 (p = 0.11, Supplementary Table S2, Fig. 3c).

Fourty-four (69%) patients entered into immunological remission at month-12 (Supplementary Table S2). There was no difference between both experimental arms (p = 0.39).

**Fig. 5:**
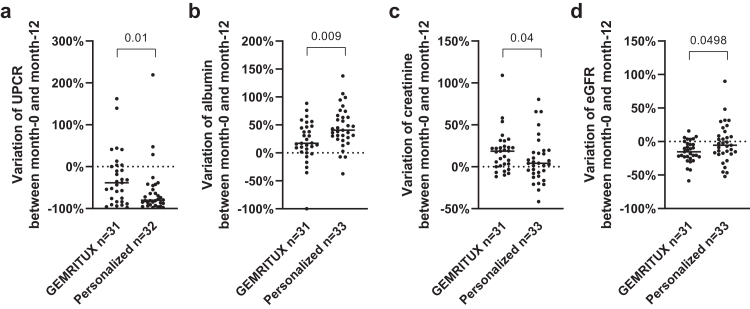
Change of clinical parameters between month-0 and month-12. a) Change of UPCR (%) between month-0 and month-12. Patients from the personalized arm had a significantly greater reduction of UPCR corresponding to improved kidney function (p = 0.01). Data was missing for one patient. b) Change of albumin (%) between month-0 and month-12. Patients from the personalized arm had a significantly greater increase of serum albumin corresponding to improved kidney function (p = 0.009). c) Change of creatinine (%) between month-0 and month-12. Patients from the personalized arm had a significantly greater reduction of serum creatinine corresponding to improved kidney function (p = 0.04). d) Change of eGFR (%) between month-0 and month-12. Patients from the personalized arm had a significantly improved eGFR (p = 0.0498). LOCF principle was used to impute missing data for all panels. LOCF, last observation carried forward; UPCR, urinary protein/creatinine ratio.

**Fig. 6:**
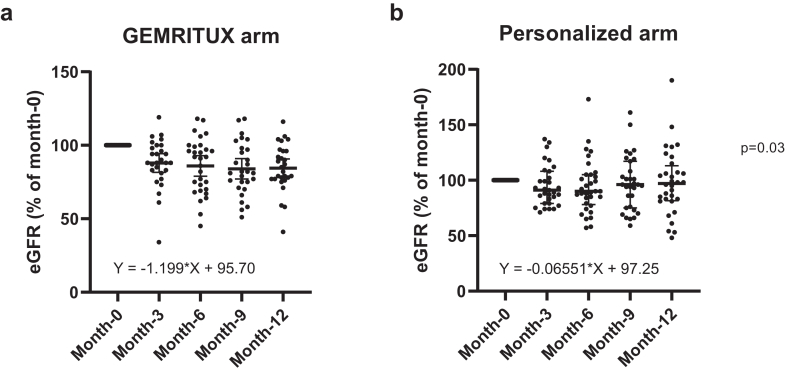
eGFR variation at each time point for GEMRITUX arm (a) and personalized arm (b). eGFR at each time point was compared to the baseline eGFR for both experimental arms. The slope of eGFR variation between month-0 and month-12 was significantly different between both arms with Y = −1.199∗X + 95.70 for the GEMRITUX arm and Y = −0.06551∗X + 97.25 for the personalized arm (p = 0.03).

The average change of UPCR, albumin, creatinine, and eGFR between month-0 and month-12 were 64% [−86%; −26%], 31% [12%; 57%], 8% [−5%; 23%], −11% [−22%; 4%], respectively, and were significantly different between the GEMRITUX and personalized arm (p = 0.01 for UPCR, p = 0.009 for albumin, p = 0.04 for creatinine, and p = 0.0498 for eGFR) (Supplementary Table S2, Fig. 5). The evolution of eGFR was significantly different between both arms (p = 0.03) (Fig. 6). Multivariable analysis performed on 54 patients who received rituximab showed that the change of eGFR at month-12 was associated with the delay between inclusion and first rituximab injection, but not with the cumulative rituximab dose received (p = 0.0004 and p = 0.06, respectively, Supplementary Table S6).

### Adverse events

Twenty-two adverse events, related or not to treatment, were reported, 10 in the GEMRITUX arm and 12 in the personalized arm (p = 0.80). Six serious adverse events were reported in each treatment arm, none related to treatment (p > 0.99, Table 3).

**Table 3:** Adverse events according to treatment group.

	All (n = 64)	GEMRITUX arm (n = 31)	Personalized arm (n = 33)	p-value
All adverse events (n, %)	22 (34%)	10 (32%)	12 (36%)	0.80
Death (n)	1	1	0	
Cardiovascular events (n)				
Ischemic stroke (n)	2	1	1	
Increased carotid stenosis (n)	1	0	1	
Carotid endarterectomy (n)	1	0	1	
Palpitations (n)	1	1	0	
Diziness with hypotension (n)	1	1	0	
Infections (n)				
Bacterial prostatitis (n)	1	0	1	
Pneumonia (n)	1	1	0	
COVID-19 with pneumonia (n)	1	0	1	
COVID-19 (n)	2	0	2	
Others (n)				
Vulvar tumor (n)	1	1	0	
Epilepsy (n)	1	0	1	
Hypo- or hyperkalemia (n)	2	2	0	
Rash and/or allergic reaction to rituximab (n)	5	1	4	
Palmar and facial erythrosis lasting three weeks after rituximab (n)	1	1	0	
Serious adverse events (n, %)	12 (9%)	6 (19%)	6 (18%)	>0.99
Serious adverse events related to treatment (n, %)	0 (0%)	0 (0%)	0 (0%)	>0.99

COVID-19: coronavirus disease 2019.

## Discussion

Sixteen years after the discovery of PLA2R1, the major autoantigen in MN, there is still no consensus on the most appropriate treatment for patients with PLA2R1-associated MN. This clinical trial demonstrates that the personalized rituximab treatment protocol based on the stratification of patients according to the immunological severity of their disease, is superior to GEMRITUX protocol in achieving clinical remission at month-12.

Stratification of patients requiring immunosuppressive treatment according to the clinical and/or immunological severity of their disease has become a vital part of treatment decision making for patients with PLA2R1-associated membranous nephropathy. Numerous studies have identified various factors that influence the probability of spontaneous remission or treatment success, including anti-PLA2R1 antibodies^9^^,^^34^, ^35^, ^36^ and PLA2R1 epitope spreading.^21^^,^^22^ KDIGO currently cites anti-PLA2R1 titer >50 RU/mL as one of the high risk factors for MN patients.^13^ Since anti-PLA2R1 titer and the presence of epitope spreading are correlated,^21^^,^^22^ this corresponds to patients with immunologically active disease, likely with epitope spreading, that are likely to see their kidney function deteriorated fast with a deposition of immune complexes on glomerular basement membrane. The value of epitope spreading as a biomarker remains debated.^20^^,^^21^^,^^23^^,^^30^^,^^37^^,^^38^ Nevertheless we show in this study that patients with epitope spreading, corresponding to patients with high anti-PLA2R1 titer in KDIGO guidelines (p = 0.007 for the association between spreading and anti-PLA2R1 titer, when patients are divided in tertiles by anti-PLA2R1 titer),^22^^,^^39^ are more likely to achieve remission in the personalized arm in which they received rituximab immediately after inclusion, than in the GEMRITUX arm, advocating for the importance of consideration of immune and not just clinical biomarkers in the treatment decision making.

In regard to the timing of immunosuppressive treatment, KDIGO currently recommends immediate treatment with immunosuppressors only for patients with life-threatening nephrotic syndrome or rapidly deteriorating kidney function.^13^ For all the remaining patients, even those at high risk, a six months wait-and-see period is recommended in order to allow patients time to enter spontaneous remission. This approach is beneficial to patients at low risk of complications and with good prognosis, who might enter into spontaneous remission and for whom immunosuppressive treatment may present unnecessary risk. If, on the other hand, the patient presents with multiple prognostic markers in the severe end of the spectrum, the patients’ kidneys may be irreversibly damaged during this wait-and-see period, rendering the subsequent immunosuppressive treatment less effective.^1^^,^^35^ In this controlled study, we aimed to give time to patients at low risk of disease progression (non-spreaders) to achieve spontaneous remission by maintaining the six months treatment with NIAT only, while the patients at high risk of disease progression (spreaders) received immediate immunosuppressive treatment. The approach proved to be beneficial since there was no difference in the rate of spontaneous remission at month-12 between both arms, demonstrating that the patients with personalized protocol were not overtreated, and since the remission rate six months after the first rituximab injection was not different between both experimental arms.

This study helps to better select patients who should benefit from early immunosuppressive treatment, and improves their quality of life since remission is achieved soon after the beginning of treatment and since the kidney function is significantly improved in comparison to the GEMRITUX protocol. The strengths of this study include its multi-center design and complete biological and immunological workup at every time point.

The study has several limitations. i) Given the complex treatment regimens, both clinicians and patients were aware of the randomization arm and the treatment allocated. However, the absence of blinding is unlikely to have an impact on study outcome, since the decision to treat and the assessment of the treatment efficacy were based on objective laboratory values. ii) A significant difference in eGFR, creatinine and BMI observed at month-0 between both treatment arms was observed in spite of randomization that was stratified neither by the participating center nor by any among the study variables. The imbalance between both arms at baseline can likely be attributed to a relatively small number of patients in each arm. The imbalance is unlikely to have affected the results of the study since all three variables had values more favorable of remission in the GEMRITUX arm. The absence of efficacy of personalized treatment in obese patients can likely be attributed either to rituximab dose and distribution volume and/or to small subgroup size. iii) We could not identify the threshold of anti-PLA2R1 antibodies over which all patients are spreaders in order to substitute epitope spreading with anti-PLA2R1 titer. If such threshold exists, it should be determined in subsequent studies with a greater number of patients. iv) In the standard arm, the GEMRITUX protocol was chosen as the reference treatment which, at the time of drafting of the study protocol, was indeed the gold standard in MN treatment.^14^ Since, it has been shown that patients receiving GEMRITUX protocol are undertreated and KDIGO now recommends 1 g rituximab infusions instead of 375 mg/m^2^.^13^ v) The standard and the experimental arm differed in three parameters: stratification according to the immunological severity of the disease or no stratification, cumulative dose of rituximab received, immediate treatment for severe patients in the personalized arm or a six months wait-and-see period for the GEMRITUX arm. While it is impossible to determine with certainty which of the three parameters contributes the most to the improved remission rates in the personalized arm, it seems that the stratification of patients and the delay before treatment, but not the rituximab dose, are responsible for better remission rates. Future clinical trials designed to compare a single variable at a time may provide a more definitive answer as to which variable contributes the most to better remission rates in the personalized arm. vi) The primary outcome of the study was evaluated 12 months after the inclusion in the study, which may be too early since patients may enter remission much later,^14^ especially in the GEMRITUX arm where patients only had six months to achieve remission after the first rituximab injection. Nevertheless, we believe it is important to treat patients using a protocol that enables them to achieve remission as early as possible to improve their quality of life and to reduce the length of sick leave. The study indeed demonstrates that a well-adapted personalized protocol can shorten time to remission.

The personalized approach to rituximab treatment based on the stratification of patients according to their PLA2R1 epitope spreading is superior to the GEMRITUX protocol in achieving the combined endpoint of partial or complete clinical remission at month-12, with a better kidney function, without reducing the rate of spontaneous remission, and with no difference in the number of adverse events. The timing and the dose of rituximab should be tailored to the clinical and immunological characteristics of each patient.

## Contributors

BS-P and VE conceived the study. VB, KZ, and CF helped with implementation. VB and KZ accessed and verified the data. KL and LB performed statistical analyses. BS-P and MT provided medical oversight. VB and BS-P wrote the manuscript. All authors contributed to refinement of the study protocol and approved the final manuscript.

## Data sharing statement

The data that support the findings of this study are available from the corresponding author upon reasonable request.

## Declaration of interests

The clinical trial was funded by DGOS (Direction Générale de l'Offre de Soins), by a grant PHRC: Clinical Research Hospital Program from the French Ministry of Health (PHRC National 2017).

Biogaran® and Celltrion Healthcare® provided Rituximab (Truxima®) at no cost for this study. Neither Biogaran® nor Celltrion Healthcare® participated in the design or conduct of the study, nor in the analysis and interpretation of the results.

BS-P and GL are co-inventors on the patent “Methods and kits for monitoring membranous nephropathy”. GL is a co-inventor on the patents “Diagnostics for membranous nephropathy” and “Profiling of immunodominant PLA2R1 epitopes as a prognosis and predictive factor in membranous nephropathy”. AL has received consulting fees and payment or honoraria for lectures, presentations, speakers bureaus, manuscript writing or educational events from CSL Vifor and AstraZeneca, and has received support for attending meetings and/or travel from CSL Vifor. VA has received support to attend meetings and/or travel from Sanofi, Genzyme, AstraZeneca, and Vifor, and has participated on a Data Safety Monitoring Board or Advisory Board for Alnylam, Vifor and AstraZeneca. All remaining authors have nothing to disclose.

## Appendix A Supplementary data

Supp Appendix clean

Protocole
