# Supplementary material for: The personalized approach to rituximab treatment in membranous nephropathy: a multi-center randomized controlled trial
Source: eClinicalMedicine. 2025 Nov 14;90:103648. doi: 10.1016/j.eclinm.2025.103648 (PMC12664402; doi:10.1016/j.eclinm.2025.103648)
Supplement: Supp Appendix clean [file mmc1.docx]

Supplementary Appendix

This appendix has been provided by the authors to give readers additional information about their work.

Supplement to: V Brglez, M Teisseyre, K Zorzi, et al. The personalized approach to rituximab treatment in membranous nephropathy: a multi-center randomized controlled trial

Table of contents of supplementary appendix

[Investigators and study coordination 3](#_Toc211590527)

[Supplementary methods 4](#_Toc211590528)

[Home-made ELISA to detect anti-PLA2R1 epitope spreading 4](#_Toc211590529)

[Supplementary figures and tables 7](#_Toc211590530)

[Supplementary Figure 1: Remission at month-12 in subgroups according to KDIGO risk 7](#_Toc211590531)

[Supplementary Figure 2: Remission at month-12 in subgroups depending on history of MN 9](#_Toc211590532)

[Supplementary Figure 3: Remission rate six months after first rituximab injection 10](#_Toc211590533)

[Supplementary Figure 4: Association between the remission rate at month-12 and the cumulative dose of rituximab received 11](#_Toc211590534)

[Supplementary Figure 5: Delay between inclusion and first rituximab infusion 12](#_Toc211590535)

[Supplementary Figure 6: Study flowchart of the per protocol analysis 13](#_Toc211590536)

[Supplementary Figure 7: Primary endpoint of partial or complete remission at month-12 as per protocol analysis 15](#_Toc211590537)

[Supplementary Table 1: Detailed analysis of epitope spreading status at baseline 16](#_Toc211590538)

[Supplementary Table 2: Outcome at month-12 17](#_Toc211590539)

[Supplementary Table 3: Outcome at month-12 for the group of patients with BMI < 30 18](#_Toc211590540)

[Supplementary Table 4: Multivariate analysis of factors influencing remission at month-12 19](#_Toc211590541)

[Supplementary Table 5: Multivariate analysis of factors influencing remission at month-12 20](#_Toc211590542)

[Supplementary Table 6: Multivariate analysis of rituximab treatment regimen factors influencing eGFR change between month-0 and month-12 21](#_Toc211590543)

[Supplementary Table 7: Specificity and positivity of epitope spreading ELISA 22](#_Toc211590544)

[Additional analyses and checklists 23](#_Toc211590545)

[Consort flow diagram 27](#_Toc211590546)

# Investigators and study coordination

The members of the PMMN study group are as follows:

**Study Investigators:**

Nice: B Seitz-Polski (Principal Investigator), M Teisseyre, M Cremoni, V Esnault

Nîmes: O Moranne, K Bijak, S Cariou, R Darmon, F Garo, M Gerbal, P Reboul

Besançon: T Crepin, A Tachikart, J Seibel

Caen: A Lanot, V Gueutin

Lyon Sud: E Novel-Catin, C Barba, L Koppe, M Massat

Bordeaux: C Rigothier

Lyon: C Pelletier, M Rozes

Paris APHP Necker: B Knebelmann, M Dao

Toulouse: D Chauveau, A Huart

Paris APHP Henri Mondor: V Audard

Tours: J-M Halimi

Avignon: D Verhelst, R André

**Study Coordination:** Délégation de la Recherche Clinique et de l’Innovation, Nice University Hospital: M Godemert

**Data Management**: Délégation de la Recherche Clinique et de l’Innovation, Nice University Hospital: E Fontas, S Cambiaggio

**Central Laboratory Coordination:** Nice University Hospital: K Zorzi, C Fernandez, V Brglez, M Godemert

**Statistical Analyses**: K Legueult, L Bailly

# Supplementary methods

## Home-made ELISA to detect anti-PLA2R1 epitope spreading

Since the analysis of epitope spreading is not commercially available, we used a home-made ELISA, as described previously.[1,2]

Generation and expression of soluble domains of PLA2R1

Full human anti-PLA2R1 protein, as well as single soluble domains CysR, CTLD1 and CTLD7 were produced as described [1,2]. The domains were generated by PCR and cloned into the pcDNA3.1Z-expression vector. They all comprised the PLA2R1 signal peptide followed by the human PLA2R1 sequence coding for the different PLA2R1 domains as follows: CysR domain (CysR, from Ala-21 to Lys-164), CTLD1 domain (CTLD1 or C1, Thr-223 to Asn-359), CTLD7 (C7, Glu-1097–1246-Leu). All soluble mutants were C-terminally HA-tagged. All domains were expressed in HEK293 cells and cell medium containing the expressed proteins was collected.

Epitope spreading ELISA

Ninety-six-well microplates were coated with anti-HA antibody (1:5,000, Sigma-Aldrich) diluted in 20 mM Tris pH 8.0 overnight at 4°C, then blocked with SeramunBlock (Seramun Diagnostica) for 2 h. The plate was then blocked with Seramun Block (Seramun Diagnostica) for 2 h, and washed three times with PBS-Tween 0.05%. PLA2R1 antigens expressed as recombinant HA-tagged proteins in the medium of HEK cells (full PLA2R1 protein (positive control for antigen), CysR, CTLD1, CTLD7,), or medium from mock-transfected HEK cells serving as negative control, were incubated in the wells for 2 h and washed. Patients' sera diluted in 0.1% (m/v) low-fat dry milk in PBS were added to wells and incubated for 2 h and washed. Care was taken to minimize freeze-thaw cycles when handling patients’ sera. Plates were then incubated for 1 h with anti-human IgG4 horseradish peroxidase (HRP)-conjugated secondary antibody (1:7,500, Southern Biotech) diluted in SeramunStab ST (Seramun Diagnostica) and washed. The signal was revealed by the addition of tetramethylbenzidine peroxidase substrate (TMB, Interchim) for 15 min before stopping the reaction with 1.2 N HCl, and the plate was read at 450 nm. The threshold of positivity of each antigen (CysR, CTLD1, and CTLD7) was determined using a ROC curve, and the value for each individual patient was corrected for the background value obtained using mock-transfected medium from HEK cells. Each plate included a standard curve for each domain, positive and negative control for antigen (full PLA2R1 protein and mock-transfected cells, respectively), as well as a positive and negative control for patients’ sera (patient who recognizes all domains, and a pool of non-MN sera, respectively). Optical density values were converted to RU/mL using a 5-parameter logistic curve (GraphPad Prism), and thresholds of positivity were determined for each antigen. Patients were considered as non-spreaders if their serum reacted only to the CysR domain, while the patients with an additional signal to either CTLD1 or CTLD7 domains or both were considered as spreaders.

The epitope spreading ELISA was set in 2018 in the Immunology laboratory at Nice University Hospital and has been used since on a regular basis (1296 sera screened thus far).

The specificity of each antigen was determined using 27 MN patients with anti-PLA2R1 antibodies >14 RU/mL according to Euroimmun anti-PLA2R1 ELISA, as well as 22 patients with nephrotic syndrome but without anti-PLA2R1 antibodies. The specificity for each individual antigen is shown in Supp Table 7. The results are line with Euroimmun’s anti-PLA2R1 ELISA that shows 100% specificity on 533 non-MN sera (manual for Euroimmun EA 1254-9601 G).

Sensitivity depends on the threshold used and is a subject of a lively debate in the MN field. For example, Reinhard et al.[3] showed that when a more sensitive technique to detect anti-domain antibodies is used, such as WB, all patients are spreaders. On the other hand, when using a less sensitive ELISA on the same cohort, only 52% and 38% of patients recognized CTLD1 and/or CTLD7 domains, respectively, similar to the original article published by Seitz-Polski et al.[2]. Other more recent studies have confirmed that an ELISA-based threshold, which identifies a lower number of spreader patients than WB-based threshold, is clinically more useful as it can predict outcome after treatment.[4–6] WB-based threshold, while more sensitive, failed to distinguish patients at risk of treatment failure.[3] While the debate whether all patients are spreaders based on a more sensitive WB or TRFIA technique is outside the scope of this manuscript, it is useful to note that more sensitivity might not be useful clinically. Nevertheless, a vast majority of studies confirm the initial results that epitope spreading is associated with worse prognosis and/or worse response to treatment,[2,4,5,7–11] while a minority of the studies do not arrive to the same conclusion.[3,6,12].

In our hands, the positivity for each antigen based on the screening of 1296 sera with anti-PLA2R1 >2 RU/mL according to Euroimmun anti-PLA2R1 ELISA, is shown in Supp Table 7. The less than 100% sensitivity for anti-PLA2R1 and anti-CysR, supposedly present in all patients, can be attributed in part to the low anti-PLA2R1 titer (based on Euroimmun kit) for some patients (between 2 and 14 RU/mL, 14 RU/mL being the official threshold of positivity) and/or to the detection method of home-made ELISA detecting IgG4 while Euroimmun commercial ELISA detects total IgG.

[1] Brglez V, Boyer-Suavet S, Zorzi K, Fernandez C, Fontas E. Personalized medicine for PLA2R1-related membranous nephropathy : A multicenter randomized control trial. Front Med 2020;7:1–11. https://doi.org/10.3389/fmed.2020.00412.

[2] Seitz-Polski B, Dolla G, Payré C, Girard C, Polidori J, Zorzi K, et al. Epitope spreading of autoantibody response to PLA2R associates with poor prognosis in membranous nephropathy. J Am Soc Nephrol 2016;27:1517–33. https://doi.org/10.1681/ASN.2014111061.

[3] Reinhard L, Zahner G, Menzel S, Koch-Nolte F, Stahl RAK, Hoxha E. Clinical relevance of domain-specific phospholipase A2 receptor 1 antibody levels in patients with membranous nephropathy. J Am Soc Nephrol 2020;31. https://doi.org/10.1681/ASN.2019030273.

[4] Seitz-Polski B, Debiec H, Rousseau A, Dahan K, Zaghrini C, Payré C, et al. Phospholipase A2 receptor 1 epitope spreading at baseline predicts reduced likelihood of remission of membranous nephropathy. J Am Soc Nephrol 2018;29:401–8. https://doi.org/10.1681/ASN.2017070734.

[5] Ghiggeri GM, Seitz-Polski B, Justino J, Zaghrini C, Payré C, Brglez V, et al. Multi-autoantibody signature and clinical outcome in membranous nephropathy. Clin J Am Soc Nephrol 2020;15:1762–76. https://doi.org/10.2215/CJN.02500220.

[6] Liu X, Xue J, Li T, Wu Q, Sheng H, Yang X, et al. Quantitative detection and prognostic value of antibodies against M-type phospholipase A2 receptor and its cysteine-rich ricin domain and C-type lectin domains 1 and 6-7-8 in patients with idiopathic membranous nephropathy. PLoS One 2024;19:1–13. https://doi.org/10.1371/journal.pone.0298269.

[7] Qin Y, Wu Q, Sheng H, Li T, Liu X, Yang X, et al. Quantitative detection of anti-PLA2R antibodies targeting different epitopes and its clinical application in primary membranous nephropathy. Clin Chem Lab Med 2022. https://doi.org/doi:10.1515/cclm-2022-0720.

[8] Zhou K, Zhou J, Zhou L, Xue J, Liu B, Zhang Z, et al. Predictive value of the domain specific PLA2R antibodies for clinical remission in patients with primary membranous nephropathy: A retrospective study. PLoS One 2024;19:1–18. https://doi.org/10.1371/journal.pone.0302100.

[9] Wu L, Su Z, Tang B, Chen Y, Hu H, Cheng Y, et al. Adverse Prognosis in Membranous Nephropathy with PLA2R1 Epitope Spreading: A Prospective Study. Am J Nephrol 2025:1–20. https://doi.org/10.1159/000545133.

[10] Zhang X, Yang F, Fan Y, Xue J, Liu B, Zhang Z, et al. Role of PLA2R domain antibodies and epitope spreading in risk stratification and prediction of proteinuria remission in primary membranous nephropathy. Sci Rep 2025;15:1–13. https://doi.org/10.1038/s41598-025-91366-5.

[11] Wu J, Zhang Q, Du Y, Zheng T, Jin J, Kao S, et al. Detection and prognostic relevance of PLA2R epitopes in idiopathic membranous nephropathy: a simultaneous quantitative multiplex suspension array detection method. Clin Kidney J 2025;18:1–11. https://doi.org/10.1093/ckj/sfaf010.

[12] Ruggenenti P, Reinhard L, Ruggiero B, Perna A, Perico L, Peracchi T, et al. Anti-Phospholipase A2 Receptor 1 and Anti-Cysteine Rich Antibodies, Domain Recognition and Rituximab Efficacy in Membranous Nephropathy: A Prospective Cohort Study. Am J Kidney Dis Off J Natl Kidney Found 2023. https://doi.org/10.1053/j.ajkd.2023.10.013.

# Supplementary figures and tables

## Supplementary Figure 1: Remission at month-12 in subgroups according to KDIGO risk

Supplementary Figure 1: Remission at month-12 in subgroups depending on KDIGO risk. a) The personalized protocol was superior to GEMRITUX protocol in achieving the combined endpoint of partial or complete remission at month-12 in the subgroup of patients with high risk disease (69% vs 37%, p=0.02), whereas there was no difference between the two arms for the patients with moderate risk disease according to KDIGO (50% vs 25%, p>0.99) (Khi-2 and Fisher test, respectively), likely due to a very small number of moderate risk patients (n=8). LOCF was used to impute missing data for remission at month-12. b) Number of patients with epitope spreading was statistically different between moderate and high risk patients according to KDIGO (p=0.005). Epitope spreading information was missing for one patient in the moderate risk group. KDIGO, Kidney Disease Improving Global Outcome; LOCF: last observation carried forward.

## Supplementary Figure 2: Remission at month-12 in subgroups depending on history of MN

Supplementary Figure 2: Remission at month-12 in subgroups depending on history of MN. Primary endpoint of partial or complete remission at month-12 for the subgroup of patients who were newly diagnosed with MN at inclusion and who had no previous immunosuppressive treatment (left panel) and for the patients included after a relapse (right panel). While there was no difference in the remission rates between the two experimental arms for relapsing patients (p=0.36), newly diagnosed patients were more likely to achieve remission if treated with personalized protocol (p=0.01). LOCF was used to impute missing data for remission at month-12 for all panels. LOCF: last observation carried forward; MN: membranous nephropathy.

## Supplementary Figure 3: Remission rate six months after first rituximab injection

Supplementary Figure 3: Remission rate six months after first rituximab injection. The combined endpoint of partial or clinical remission was assessed at the follow-up visit six months after the first injection, for the subset of patients who were treated with either low or high dose rituximab. There was no difference in the remission rate between both arms (p>0.99). Data was missing for one patient in each arm who received rituximab but were lost to follow-up before their visit six months post rituximab. LOCF was used to impute missing data for remission at month-12 for all panels. LOCF: last observation carried forward.

## Supplementary Figure 4: Association between the remission rate at month-12 and the cumulative dose of rituximab received

Supplementary Figure 4: Association between the remission rate at month-12 and the cumulative dose of rituximab received. a) Association between the remission rate at month-12 and the cumulative dose of rituximab received for the entire cohort, both arms combined. There was no difference in the cumulative dose of rituximab between the patients who achieved the combined endpoint or not (p=0.32). b) Association between the remission rate at month-12 and the cumulative dose of rituximab received in GEMRITUX (left panel) and personalized arm (right panel). There was no difference in the cumulative dose of rituximab between the patients who achieved the combined endpoint or not (p=0.32 and p=0.68 for GEMRITUX and personalized arm, respectively). LOCF was used to impute missing data for remission at month-12 for all panels. LOCF: last observation carried forward.

## Supplementary Figure 5: Delay between inclusion and first rituximab infusion

Supplementary Figure 5: Delay between inclusion and first rituximab injection. There was a statistically different delay between inclusion and first rituximab injection in both experimental arms (p=0.0004) in the subgroup of patients treated with rituximab. Date of first rituximab injection was missing for one patient.

## Supplementary Figure 6: Study flowchart of the per protocol analysis


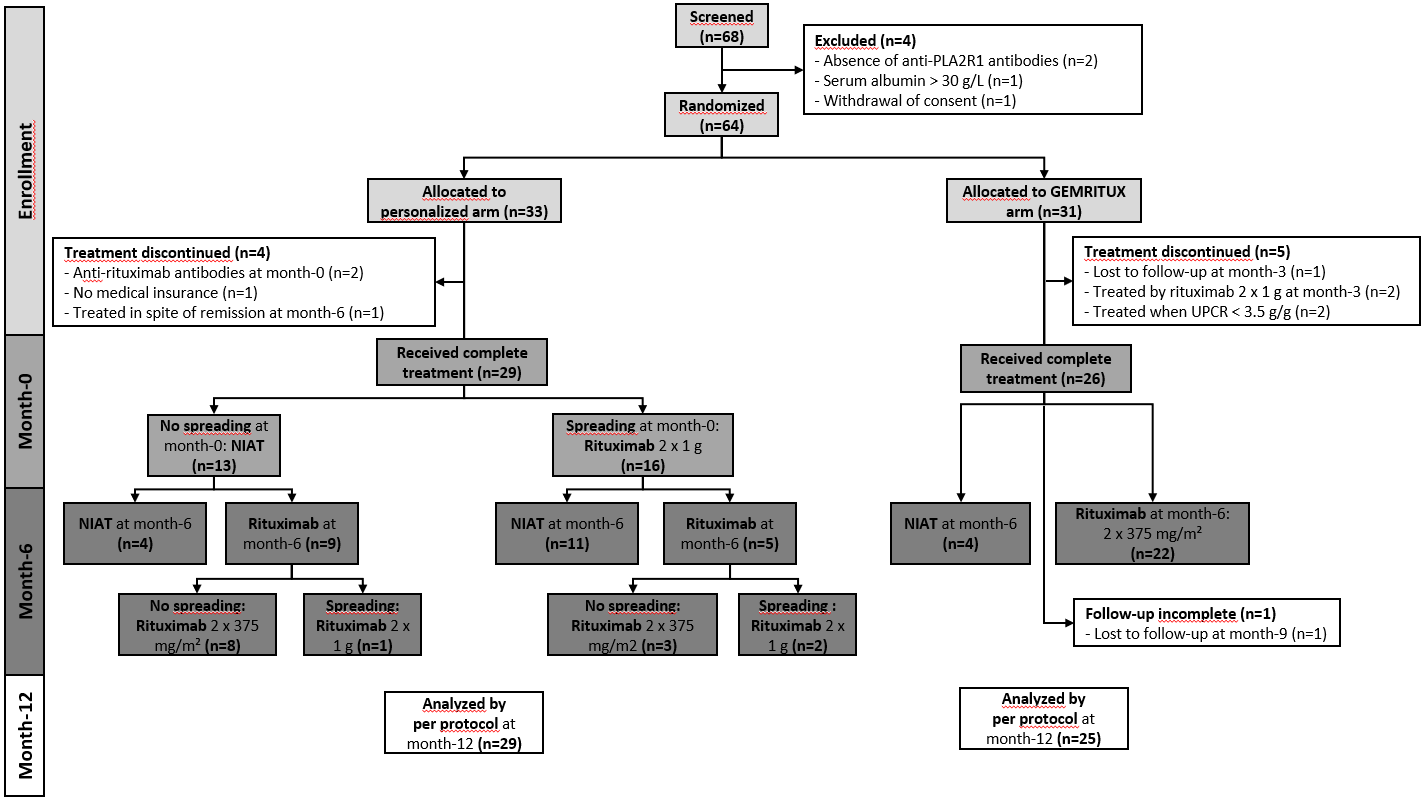


Supplementary Figure 6: Study flowchart of the per protocol analysis. From the 68 patients screened, 64 were included. Thirty-three (52%) included patients were randomized in the personalized arm, and 31 (48%) in the GEMRITUX arm. Ten patients (16%) deviated from the protocol. One patient (2%) was erroneously included in the study due to the lack of medical insurance. Two relapsing patients (3%) were erroneously included due to the presence of anti-rituximab antibodies. One patient was treated with rituximab following the protocol, while the second patient received obinutuzumab. Two patients (3%) from the GEMRITUX arm were lost to follow-up before month-12, one before receiving any treatment and the other at month-9 after having received complete treatment. Five patients (8%) were not treated according to the protocol: two patients in the GEMRITUX arm were treated prematurely at month-3 since their symptoms worsened, and three patients from both GEMRITUX (n=2) and personalized arm (n=1) were treated at month-6 in spite of not fulfilling the treatment criteria (UPCR < 3.5 g/g). Twenty-nine patients from the personalized arm and 26 patients from the GEMRITUX arm received complete treatment. Patients from the GEMRITUX arm received NIAT only for six months. At month-6, either the patients achieved spontaneous remission (n=4), in which case NIAT only was continued, or they had an active disease (n=22), in which case they were treated with two 375 mg/m^2^ rituximab injections at one week interval. Patients from the personalized arm were stratified at month-0 according their epitope spreading status. Patients without epitope spreading (n=13) received NIAT only for six months. Patients with epitope spreading (n=16) were treated immediately with two 1 g rituximab injections at two weeks interval. The clinical and immunological profile of patients in the personalized arm was reassessed at month-6. Patients in spontaneous (n=4) and rituximab-induced remission (n=11) received NIAT only. Patients with active disease and without epitope spreading at month-6 were treated (n=8 for patients without epitope spreading at month-0) or retreated (n=3 for patients with epitope spreading at month-0 who have already received rituximab at month-0) with two 375 mg/m^2^ rituximab injections at one week interval. Patients with active disease and with epitope spreading at month-6 were treated (n=1 for patients without epitope spreading at month-0) or retreated (n=2 for patients with epitope spreading at month-0 who have already received rituximab at month-0) with two 1 g rituximab injections at two weeks interval. Twenty-nine patients from personalized arm and 25 patients from GEMRITUX arm were included in the final per protocol analysis. NIAT: non-immunosuppressive antiproteinuric treatment; PLA2R1, phospholipase A2 receptor 1.

## Supplementary Figure 7: Primary endpoint of partial or complete remission at month-12 as per protocol analysis

Supplementary Figure 7: Primary endpoint of partial or complete remission at month-12 as per protocol analysis on a subset of 54 patients without deviation from the protocol. Nine patients (36%) from GEMRITUX arm achieved the composite endpoint of partial or complete remission at month-12, versus 20 (69%) from the personalized arm (p=0.02).

## Supplementary Table 1: Detailed analysis of epitope spreading status at baseline

Supplementary Table 1: Detailed analysis of epitope spreading status at baseline

|  | **All (n=64)** |
| --- | --- |
| **Epitope spreading (n, %)** | 32 (51%)^a^ |
| **Positive for anti-CysR (n, %)** | 63 (100%)^a^ |
| **Positive for anti-CTLD1 (n, %)** | 22 (35%)^a^ |
| **Positive for anti-CTLD7 (n, %)** | 19 (30%)^a^ |
| **Epitope spreading profile group (n, %)** | Anti-CysR only: 32 (51%)^a^  Anti-CysR + anti-CTLD1: 12 (19%)  Anti-CysR + anti-CTLD7: 9 (14%)  Anti-CysR + anti-CTLD1 + anti-CTLD7: 10 (16%) |
| **Anti-CysR titer (RU/mL)** | 53 [33; 241]^a^ |
| **Anti-CTLD1 titer for patients positive for anti-CTLD1 (RU/mL)** | 290 [88; 1770]^a^ |
| **Anti-CTLD7 titer for patients positive for anti-CTLD7 (RU/mL)** | 172 [76; 312]^a^ |

^a^ Data on epitope spreading was missing for one patient

## Supplementary Table 2: Outcome at month-12

Supplementary Table 2: Outcome at month-12

|  | **All (n=64)** | **GEMRITUX arm (n=31)** | **Personalized arm (n=33)** | **P value** |
| --- | --- | --- | --- | --- |
| **Combined endpoint of partial or complete clinical remission (n, %)^a^** | 33 (52%) | 11 (35%) | 22 (67%) | **0.01** |
| Spontaneous remission (n, %)^b^ | 5 (15%) | 2 (18%) | 3 (14%) | >0.99 |
| **Complete clinical remission (n, %)^b^** | 4 (6%) | 0 (0%) | 4 (12%) | 0.11 |
| **Immunological remission (n, %)^b^** | 44 (69%) | 19 (61%) | 25 (76%) | 0.39 |
| **Change of anti-PLA2R1 (%)^b,c,d^** | -90 [-97; -72]^c^ | -89 [-96; -78]^e^ | -93 [-98; -58]^e^ | 0.25 |
| **Change of creatinine (%)^b^** | 8 [-5; 23] | 19 [1; 24] | 4 [-10; 18] | **0.04** |
| **Change of eGFR (%)^b^** | -11 [-22; 4] | -15 [-23; -3] | -5 [-18; 12] | **0.0498** |
| **Change of albumin (%)^b^** | 31 [12; 57] | 17 [2; 46] | 41 [26; 65] | **0.009** |
| **Change of UPCR (%)^b,e^** | -64 [-86; -26]^e^ | -39 [-84; 4] | -80 [-90; -48]^e^ | **0.01** |

eGFR: estimated glomerular filtration rate; PLA2R1: phospholipase A2 receptor; UPCR: urinary protein/creatinine ratio

^a^ Worst-case scenario was used to impute missing data.

^b^ LOCF was used to impute missing data.

^c^ Missing values for two patients.

^d^ Patients without detectable anti-PLA2R1 were assigned 2 RU/mL to calculate change between month-0 and month-12.

^e^ Missing value for one patient.

## Supplementary Table 3: Outcome at month-12 for the group of patients with BMI < 30

Supplementary Table 3: Outcome at month-12 for the group of patients with BMI < 30

|  | **All (n=49)** | **GEMRITUX (n=27)** | **Personalized (n=22)** | **P value** |
| --- | --- | --- | --- | --- |
| Combined endpoint of partial or complete clinical remission (n, %) | 27 (55%) | 10 (37%) | 17 (77%) | **0.009** |
| Complete clinical remission (n, %) | 3 (6%) | 0 (0%) | 3 (14%) | 0.08 |
| Immunological remission (n, %) | 35 (71%) | 18 (67%) | 17 (77%) | 0.53 |

## Supplementary Table 4: Multivariate analysis of factors influencing remission at month-12

Supplementary Table 4: Multivariate analysis of factors influencing remission at month-12

|  | **p univariate analysis** | **p multivariate analysis** | **Odds ratio** | **95% confidence interval** |
| --- | --- | --- | --- | --- |
| Treatment arm (GEMRITUX/Personalized) | 0.013 | 0.003 | 7.229 | [1.942 - 26.901] |
| BMI (kg/m^2^) | 0.023 | 0.013 | 0.840 | [0.732 - 0.964] |
| eGFR (mL/min/1.73 m^2^) | 0.028 | 0.96 | 1.001 | [0.975 - 1.026] |

## Supplementary Table 5: Multivariate analysis of factors influencing remission at month-12

Supplementary Table 5: Multivariate analysis of rituximab treatment regimen factors influencing remission at month-12

|  | **p univariate analysis** | **p multivariate analysis** | **Odds ratio** | **95% confidence interval** |
| --- | --- | --- | --- | --- |
| Delay between inclusion and 1^st^ rituximab dose (days) | 0.0004 | 0.002 | 0.981 | [0.969-0.993] |
| Cumulative rituximab dose (mg) | 0.32 | 0.15 | 0.999 | [0.998-1.000] |

## Supplementary Table 6: Multivariate analysis of rituximab treatment regimen factors influencing eGFR change between month-0 and month-12

Table 6: Multivariate analysis of rituximab treatment regimen factors influencing eGFR change between month-0 and month-12

|  | **p univariate analysis** | **p multivariate analysis** | **Estimates** |
| --- | --- | --- | --- |
| Delay between inclusion and 1^st^ rituximab dose (days) | 0.0021 | 0.0004 | -0.16841 |
| Cumulative rituximab dose (mg) | 0.6554 | 0.0630 | -0.01157 |

## Supplementary Table 7: Specificity and positivity of epitope spreading ELISA

Supplementary Table 7: Specificity and positivity of epitope spreading ELISA

|  | **Specificity (%)** | **Positivity (%)** |
| --- | --- | --- |
| Full PLA2R1 | 100% | 95% |
| CysR | 95% | 94% |
| CTLD1 | 100% | 33% |
| CTLD7 | 100% | 23% |

# Additional analyses and checklists

## Consort flow diagram

**CONSORT 2010 Flow Diagram**

Enrollment

**Analysed (n=31)**
♦ Primary outcome data complete (n=29)

♦ Primary outcome data incomplete, considered success (n=2)

**Excluded from analysis (n=0)**

**Analysed (n=33)**
♦ Primary outcome data complete (n=33)

♦ Primary outcome data incomplete, considered failure (n=0)

**Excluded from analysis (n=0)**

Lost to follow-up before month-12 (n=0)

Discontinued intervention (n=0)

Lost to follow-up before month-12 (n=2)

♦ Lost to follow-up (n=2)

Discontinued intervention (n=0)

Follow-Up

**Allocated to Personalized arm (n=33)**

♦ Received allocated intervention (n=29)

♦ Did not receive allocated intervention (n=4)

- Anti-rituximab antibodies at month-0 (n=2)
- No medical insurance (n=1)
- Treated in spite of remission at month-6 (n=1)

Excluded (n=4)

♦  Absence of anti-PLA2R1 antibodies (n=2)

♦  Serum albumin > 30 g/L (n=1)

♦  Withdrawal of consent (n=1)

Allocation

Randomized (n=64)

Assessed for eligibility (n=68)

Analysis

**Allocated to GEMRITUX arm (n=31)**

♦ Received allocated intervention (n=26)

♦ Did not receive allocated intervention (n=5)

- Lost to follow up at month-3 (n=1)
- Treated by rituximab 2 x 1 g at month-3 (n=2)
- Treated when UPCR < 3.5 g/g (n=2)
