# Supplementary material for: The personalized approach to rituximab treatment in membranous nephropathy: a multi-center randomized controlled trial
Source: eClinicalMedicine. 2025 Nov 14;90:103648. doi: 10.1016/j.eclinm.2025.103648 (PMC12664402; doi:10.1016/j.eclinm.2025.103648)
Supplement: Protocole [file mmc2.pdf]

***Personalized Medicine for Membranous Nephropathy***

***PMMN***

N°EudraCT : 2018-002476-40 – N° Interne : 17-APN-01

**Version 4.0 du 28/04/2023**


**PROMOTEUR**

CHU de Nice

Délégation à la Recherche Clinique et à l'Innovation

Hôpital de Cimiez

4, av Reine Victoria

CS 91179 06003 Nice cedex 01

Tel: 04 92 03 40 11 – Fax: 04 92 03 40 75

drc@chu-nice.fr

**INVESTIGATEUR COORDONNATEUR**

Barbara SEITZ-POLSKI

Néphrologie et Immunologie

CHU de Nice

Hôpital l'Archet I

BP 3079, Nice Cedex 03

Tel : 04 92 03 55 02 - Fax : 04 92 03 54 70

e-mail: seitz-polski.b@chu-nice.fr

**SOMMAIRE**

SUMMARY ....................................................................................................... 4
SCIENTIFIC RATIONALE .................................................................................... 4
1. GENERAL DESCRIPTION AND SCIENTIFIC RATIONALE ................................. 8
2. STUDY OBJECTIVES ..................................................................................... 14
2.1 PRIMARY OBJECTIVE ................................................................................. 14
2.2 SECONDARY OBJECTIVES .......................................................................... 15
3. EVALUATION CRITERIA ............................................................................... 15
4. TYPE OF STUDY ........................................................................................... 17
5. BENEFIT/ RISK .............................................................................................. 17
*Benefit* ............................................................................................................ *17*
- *At the individual level:* .............................................................................. *17*
- *At the collective level:* ............................................................................... *17*
*Risk* ................................................................................................................. *17*
*Conclusion: All things considered, the risk/benefit balance appears to be favorable in this study.* ..................................................................................................... *18*
6. SELECTION AND EXCLUSION CRITERIA FOR STUDY PARTICIPANTS ............... 18
6.1 INCLUSION CRITERIA ................................................................................. 18
6.2 EXCLUSION CRITERIA ................................................................................. 19
7. SAMPLE SIZE CALCULATION ........................................................................ 19
8. DESCRIPTION OF THE CONDUCT OF THE STUDY ........................................... 20
9. TREATMENT ................................................................................................. 29
**9.1 STUDY TREATMENT** ..................................................................................... 29
***- Investigational Product*** ........................................................................... *29*
***- Packaging and Labeling*** .......................................................................... *30*
***- Handling and Dispensing*** ........................................................................ *30*
**9.3 CONCOMITANT TREATMENTS** ...................................................................... 31
***- Prohibited and/or Restricted Treatments*** ................................................ *31*
***- Permitted therapy*** .................................................................................... *31*
10. REPORTING ADVERSE EVENTS (AES) AND SERIOUS ADVERSE EVENTS (SAES) ...................... 32
10.1 DEFINITIONS ............................................................................................. 32
10.2 CAUSALITY ................................................................................................ 33
10.3 ADVERSE EVENT REPORTING ..................................................................... 34
10.4 ASSOCIATION WITH THE STUDY DRUG ........................................................ 35

10.5 SAE AND SUSAR REPORTING PROCEDURE ........ 35
10.6 REPORTING OF SUSPECTED UNEXPECTED SERIOUS ADVERSE REACTIONS BY THE SPONSOR TO THE AGENCY ........ 36
10.7 EXCEPTION RULES FOR SAE REPORTING ........ 37
10.8 EXPECTED ADVERSE EVENTS ........ 38
10.9 ANNUAL REPORTING BY THE SPONSOR TO THE AGENCY ........ 43
10.10 WITHDRAWAL OF STUDY PARTICIPANTS ........ 44
10.11 PREGNANCY ........ 44
10.12 DATA SAFETY MONITORING BOARD (DSMB) ........ 45
11. DATA COLLECTION ........ 45
12. STATISTICAL ANALYSIS ........ 46
13. FEASIBILITY ........ 48
15. STUDY RESOURCES ........ 49
16. ETHICAL AND REGULATORY REQUIREMENTS: ........ 50
17. RULES GOVERNING PUBLICATION ........ 55

## SUMMARY

| Title |
|---|
| Personalized Medicine for Membranous Nephropathy |
| Sponsor |
| CHU de Nice |
| Principal Investigator |
| Barbara SEITZ-POLSKI, Néphrologie et Immunologie, CHU de Nice |
| Associated Investigators (*Centre de Référence Maladies Rares « Syndrome Néphrotique »*) |
| Vincent AUDARD, Néphrologie, AP-HP<br>Vincent ESNAULT, Néphrologie, CHU de Nice<br>Bertrand KNEBELMANN, Néphrologie, AP-HP<br>Gérard LAMBEAU, CNRS, IPMC, Sophia-Antipolis<br>Dil SAHALI, INSERM, AP-HP |
| |
| |
| Pathology: |
| Idiopathic Membranous Nephropathy (iMN) related to PLA2R1-antibodies with a nephrotic syndrome |
| Scientific rationale |
| Our recent works on the characterization of PLA2R1 epitopes in iMN and on the comparison of different protocols of rituximab dosing showed that:<br>- ***epitope spreading at baseline should be considered for early therapeutic intervention***<br>- ***early remission rate depends on rituximab dosing,***<br>- ***low dose of rituximab could be enough for patients with anti-PLA2R1 activity restricted to CysR,***<br>- ***high dose of rituximab may be required for patients with epitope spreading.*** |
| Primary and secondary objectives: |
| <u>*Primary objectives:*</u><br>To compare the efficacy of a ***personalized treatment*** of nephrotic iMN driven by anti-PLA2R1 antibody epitope profile at month 0 and month 6 with the ***GEMRITUX*** therapeutic ***protocol*** to induce clinical remission of the nephrotic syndrome at month-12.<br><u>*Secondary objectives:*</u><br>To compare between a personalized treatment of iMN driven by anti-PLA2R1 antibody epitope profiles and the *GEMRITUX* therapeutic protocol:<br>- Complete clinical and immunological remissions at M6, M9, M12, M18, M24<br>- Partial clinical remission at M6, M9, M12, M18, M24<br>- Proteinuria and albuminuria at M6, M9, M12, M18, M24<br>- Changes in proteinuria and albuminuria from baseline at M6, M9, M12, M18, M24<br>- Serum creatinine and estimated glomerular filtration rate (eGFR) at M6, M9, M12, M18<br>- Changes in serum creatinine and eGFR from baseline at M6, M9, M12 and M18<br>- PLA2R1-Ab titers at M6, M9, M12, M18 and M24<br>- Serious adverse<br><br>The following objectives aim to study risk factors for remission or relapse:<br>- To determine risk factors for remission (immunological and clinical, partial or complete) at M12 in patients pooled from the two arms |

| | |
|---|---|
| To determine risk factors for relapse (between M12 and M24) in patients responders at M12, pooled from the two arms | |

| Population |
|---|
| n= 64 iMN patients with nephrotic syndrome and anti-PLA2R1 antibodies |
| Study duration |
| Inclusion 4 years, follow-up 2 years, total duration 6 years |
| Experimental plan |

Randomized, open label, multicentre (22 centres), prospective trial comparing the efficacy of two therapeutic strategies to obtain clinical remission 1 year after diagnosis of iMN with nephrotic syndrome and anti-PLA2R1 antibodies:

- GEMRITUX protocol: 6 months of symptomatic antihypertensive and antiproteinuric therapy, and if the nephrotic syndrome persists at month-6 (urinary protein/creatinine ratio (UPCR) remains > 3.5 g/g and albuminemia < 30 g/l), two 375 mg/m$^2$ rituximab infusions at 1-week interval.
- Personalized treatment:
  - restricted anti-CysR activity at inclusion: 6-month symptomatic antihypertensive and antiproteinuric treatment (KDIGO)
  - restricted anti-CysR activity after 6 months of symptomatic treatment with persisting nephrotic syndrome (UPCR remains > 3.5 g/g and albuminemia < 30 g/l): two 375 mg/m$^2$ rituximab infusions at 1-week interval;
  - Anti-CTLD1/7 activity at inclusion or after 6 months with persisting nephrotic syndrome (UPCR remains > 3.5 g/g and albuminemia < 30 g/l): two 1g rituximab infusions at 2-week interval at month 0 and/or month 6. For patients who will develop anti-rituximab antibodies, treatment with human anti-CD20 is widely recommended.

Selection criteria

Inclusion criteria:

- Age 18 years or more
- Anti-PLA2R1 activity detected by ELISA or Euroimmune IFA
- Nephrotic syndrome defined by proteinuria > 3.5 g/24h (or UPCR > 3.5 g/g) and serum albumin < 30 g/L at diagnosis
- eGFR (CKD-EPI) > 30 ml/min/1,73 m$^2$ at diagnosis
- Symptomatic treatment according to KDIGO guidelines: maximal tolerated dose of NIAT (angiotensin-converting enzyme inhibitor and/or angiotensin 2 receptor blockers, diuretics and statins)
- Medical insurance
- Signed informed consent
- Having understood and accepted the need for long-term medical follow-up
- Woman of child-bearing age must be using an effective method of contraception

Exclusion criteria:

- Secondary MN: MN related to cancer, infectious, systemic lupus erythematosis, drug
- Anti-PLA2R1 antibodies not confirmed by central analysis (in this case the patient will be replaced)
- Pregnancy or breastfeeding
- Immunosuppressive treatment in the 6 last months
- Presence of anti-rituximab antibodies for relapsed patients

| |
|---|
| - Cancer under treatment<br>- Patient with complicated nephrotic syndrome that would require early immunosuppressive treatment (thrombosis, acute renal failure…)<br>- Patients with active, severe infections or active hepatitis B<br>- Hypersensitivity to the active substance or to murine proteins, or to any of the other excipients<br>- Patients in a severely immunocompromised state<br>- Severe heart failure (New York Heart Association Class IV) or severe, uncontrolled cardiac disease<br>- Patients unable to give an informed consent |
| Description of the conduct study |
| Patients with clinical diagnosis of MN will be tested for the presence of PLA2R1-Ab according to local practices. The selection of the patients fulfilling the inclusion criteria will not delay the start of the symptomatic treatment according to KDIGO guidelines. If the patient meets the criteria for enrollment in the study, they will be informed of the study by the referring nephrologist. Full information will be supplied orally, together with written information. After signing informed consent, blood samples will be shipped to Nice University hospital to analyze the anti-PLA2R1 epitope profiles and anti-rituximab antibodies. Once confirmation of inclusion criteria, patients will be randomized 1/1 between conventional and personalized treatments. All patients will be re-evaluated post-treatment (symptomatic or rituximab) at M3, M6, M9, M12, M18 and M24, with centrally analyzed anti-PLA2R1 activity and epitope profile. |
| Principal criteria |
| Clinical remission at month-12 (KDIGO definition):<br>- complete: UPCR <0.3 g/g in spot morning urine samples and Normal serum albumin and eGFR > 60 ml/min/1.73 $m^2$<br>- partial: UPCR < 3.5 g/g with a decrease greater than 50% from baseline and improvement or normalization of the serum albumin concentration and increase of serum creatinine lower than 20% |
| Methodology and biostatistics |
| Biometry department of the *Delegation à la Recherche Clinique et à l'Innovation* of Nice University Hospital |

***Abbreviations used in this document***

MN : Membranous Nephropathy

iMN : idiopathic Membranous Nephropathy

NEP : Neutral Endopeptidase

PLA2R1 : m-type phospholipase A2 receptor

THSD7A : Thrombospondin type-1 domain containing 7A

KDIGO : Kidney Disease Improving Global Outcome

ELISA : Enzyme Linked Immunosorbent Assay

IFA : Immunofluorescence Assay

CD20 : Cluster Differenciation 20

NIAT : Non immunosuppressive antiproteinuric treatment

CTLD : C-type lectin domain

CysR : Cysteine-rich domain

eGFR : estimate glomerular filtration rate

CKD-EPI: Chronic Kidney Disease-EPIdemiology collaboration

UPCR : Urinary protein/creatine ratio

UACR : Urinary albumin/creatine ratio

TReg : Lymphocyte T regulator

HBC : Hepatits B virus

HCV : Hepatitis C virus

eCRF : electronic case report file

IL-35 : Interleukin 35

LOCF : Last Observation carried forward

Ab : antibodies

GEMRITUX protocol: 6 months of symptomatic antihypertensive and antiproteinuric therapy, and if the nephrotic syndrome persists at month-6 (UPCR remains > 3.5 g/g and albuminemia < 30 g/l), treatment with two 375 mg/$m^2$ infusions at 1-week interval.

# 1. General description and scientific rationale

## 1. 1 Current context

Membranous nephropathy (MN) is a rare disease with an incidence of 1,3 cases/ 100 000 people/year in France (1) but it can be severe: the risk of end stage kidney disease involves 1 out of 5 male and 1 out of 12 females, 15% of patients with MN were at risk to reach end stage kidney disease after at least 10 years of disease evolution. It is the first cause of nephrotic syndrome in adults (2, 3).

MN is defined by subepithelial immune deposits containing IgG with alteration of the membrane structure. MN can be idiopathic (or primary) without any identified cause (70-80% of cases), or secondary to clinical disorders such as hepatitis B, systematic lupus erythematosus, cancer and drug side effect (4, 5). Idiopathic MN (iMN) is an autoimmune disease directed against a podocyte antigen, such as neutral endopeptidase (NEP) in the neonate, M-type phospholipase A2 receptor (PLA2R1) and thrombospondin type-1 domain-containing 7A (THSD7A) in 70-80% and <5% adult patients respectively (6-8). The pathogenic role of PLA2R1-antibodies (Ab) is not yet proven, but antibody titers usually rise during clinically active phases and decrease before normalization of proteinuria (9, 10). Spontaneous remission occurs in about one third of patients, and kidney failure in another third (11, 12). High titers of PLA2R1-Ab at presentation and their persistence predict poor clinical outcome (13, 14). Therefore, reducing PLA2R1-Ab levels has become an important goal of therapy.

The treatment of iMN is controversial. Kidney Disease Improving Global Outcomes (KDIGO) guidelines recommend a supportive symptomatic treatment with blockers of the renin-angiotensin system and diuretics in all patients with iMN, and immunosuppressive therapy only in the case of renal function deterioration or persistent nephrotic syndrome. Therefore, immunosuppressive treatments are often started only after significant and potentially irreversible complications. On the other hand, an unnecessarily early start of immunosuppression can be futile in patients who develop remission with symptomatic treatments. Therefore, there is a need for better predictors of renal outcome in iMN.

KDIGO guidelines recommend an initial immunosuppressive therapy with a 6-month course of alternating monthly cycles of oral and intravenous corticosteroids and oral alkylating agent (cyclophosphamide rather than chlorambucil for initial therapy) (recommendation grade 1B and 2B). Cyclosporine or tacrolimus can be used as alternative regimens for the initial therapy

(recommendation grade 1C). All these immunosuppressive treatments are associated with side effects (15).

The anti-CD20 antibody (rituximab) has been increasingly used off-label in iMN, like in other B-cell dependent diseases with a low rate of side effects. Rituximab can trigger B-cell death and induce PLA2R1-Ab depletion and clinical remission in 60-80% of patients with iMN in several non-randomized study (9, 16, 17). Its long-term efficacy was established in a recent randomized controlled study (GEMRITUX). Seventy-five iMN patients (PLA2R1-Ab+ n=58, and PLA2R1-Ab- n=17 using IFA) with nephrotic syndrome after 6-months therapy with non-immunosuppressive antiproteinuric treatment (NIAT) were randomly allocated to 375mg/m$^2$ intravenous rituximab plus NIAT on days 1 and 8 (n=37) or NIAT alone (n=38). After 6 months, remission rate (35.1% in rituximab group vs 21.1% in NIAT alone group) was not different between the 2 groups (p=0.21), while remission rate after a median time of 17 months during observational phase were higher in the rituximab group (64.9% vs 34.2% respectively p<0.01) (18). The negative results of this study at 6 months raise the question of the **selection of patients that received immunosuppressive therapy** and of the **dose of rituximab used**.

### 1.2 Epitope Spreading as prognosis biomarker

Our first objective is to better select patients who will benefit from immunosuppressive treatment. Although the identification of PLA2R1-Ab has been paradigm shifting in the diagnosis and management of patients, there are outliers who call for additional biomarkers. Indeed, antibodies may persist during apparent clinical remission and conversely a drop in antibody titer may not be associated with a clinical remission (14, 19, 20).

PLA2R1 is a 180-kDa membrane receptor with a large extracellular region comprising 10 distinct globular domains including a cysteine-rich domain (CysR), a fibronectin type II domain (FNII), and 8 distinct C-type lectin domains (CTLD1-8) (21). Each domain is separated by a small linker sequence of about 10 amino acids. An immunodominant epitope was first identified in a region spanning the CysR-FNII-CTLD1 domains (22), which was further restricted to the CysR domain alone (23). In a first study, we identified reactive epitopes in the CysR, CTLD1 and CTLD7 domains, and that all samples reacted with CysR domain confirming that CysR was the immunodominant epitope. We further showed that patients with anti-CysR-restricted activity were younger, had lower proteinuria, and exhibited a higher rate of spontaneous remission and lower rates of renal failure progression (24). Conversely, high PLA2R1-Ab activity and epitope spreading beyond the CysR epitope were

independent risk factors of poor renal prognosis in a collection of 69 sera from five French nephrology centers.

In a second prospective trial we analyzed the predictive value of epitope-specific PLA2R1-Ab titer and epitope spreading at diagnosis in the 58 PLA2R1-Ab positive patients from GEMRITUX randomly allocated to rituximab plus NIAT (n=29) or NIAT (n=29). In adjusted analysis, epitope spreading at baseline was associated with a decreased remission rate at month 6 (OR, 0.16; 95% CI, 0.035 to 0.72; p=0.0171) and last follow-up (median, 23 months; OR, 0.14; 95% CI, 0.03 to 0.64; p=0.0107), independently from age, gender, baseline PLA2R1-Ab level and treatment group (Seitz-Polski et al. JASN Accepted august 2017).

Our findings led us to hypothesize that PLA2R1-Ab may be initially raised against the N-terminal CysR domain with pauci-symptomatic iMN disease: patients in the CysR group are younger than in the others groups, suggesting that these patients are probably at the beginning of the disease and should entered in spontaneous remission. Some patients could be asymptomatic at this stage. A second immune challenge (allergy, infection…) might then induce intramolecular spreading in PLA2R1 toward the C-terminal end (CTLD1 or CTLD7) leading to more active disease.

**Figure 1: MN sera exhibit 1, 2 or 3 distinct antibodies targeting different PLA2R1 domains Working hypothesis of natural history: 1. CysR as the first immunodominant epitope. 2. Epitope spreading towards CTLD1 and CTLD7**

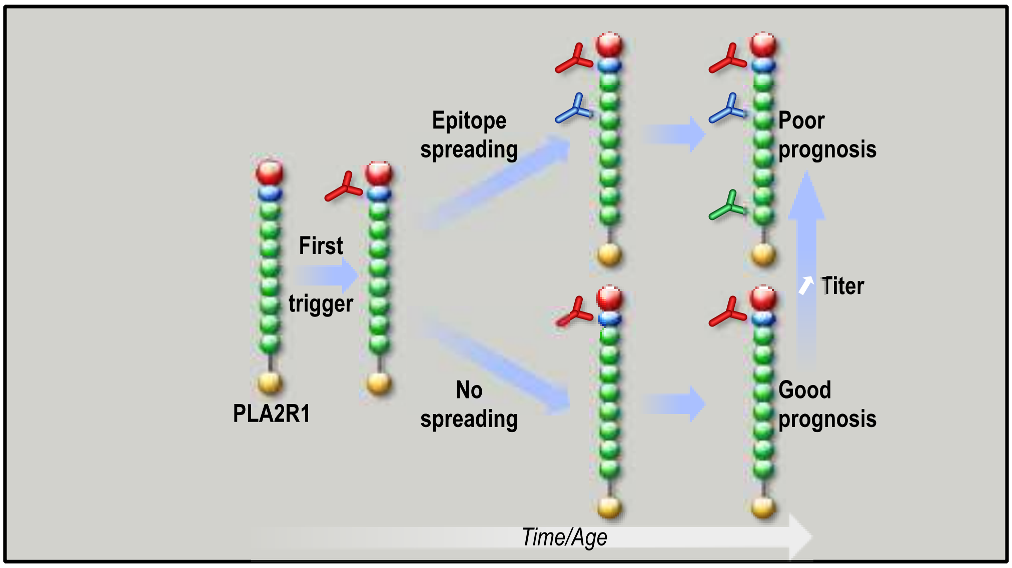


***We conclude that epitope spreading at baseline should be considered for early therapeutic intervention in patients with iMN.***

**Figure 2: Monitoring of epitope spreading as a clinical biomarker**

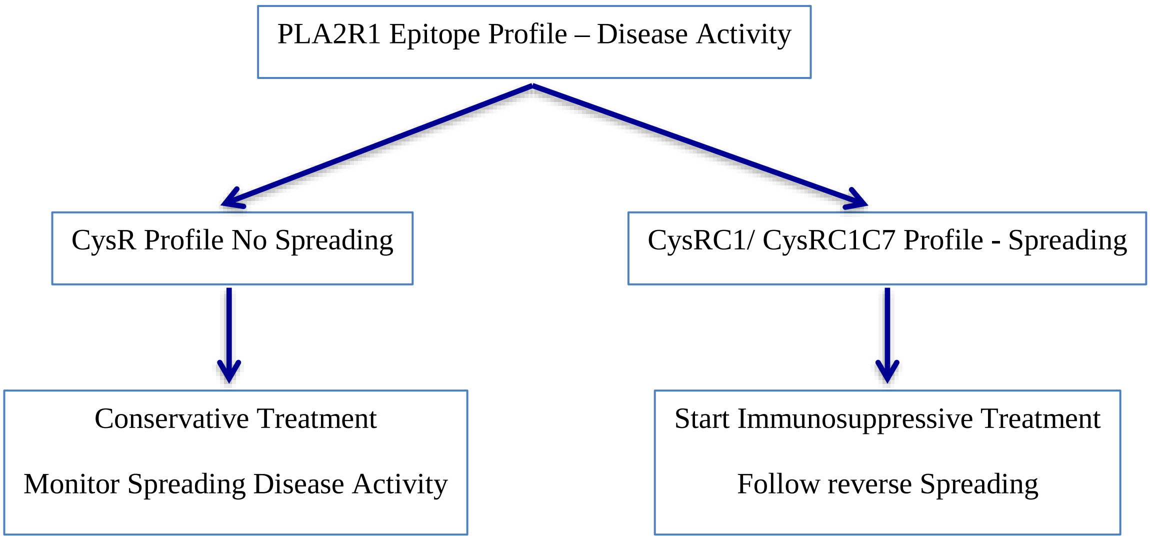


**1.3 Rituximab protocol**

Our second objective is to adapt the dose of rituximab to the immunological activity of the disease. There are still uncertainties regarding the protocol of rituximab that should be used in heavily nephrotic patients (25). Cravedi et al. proposed to titrate rituximab to CD20 cell counts (26). Fervenza et al. showed that four doses of rituximab resulted in more effective B-cell depletion, but proteinuria reduction was similar in patients who received 2 doses of 1g at one-week interval (27). Pharmacokinetic studies have demonstrated a large inter-individual variability, related to either disease or genetic factors, which could explain differences in clinical response (28-31). Residual rituximab serum levels were detected in some patients up to 6 to 9 months after the first infusion due to recycling from endothelial cells via FcRn receptor. In patients with MN, rituximab half-life was calculated as 11.5 days, compared to 18.0 days in patients with rheumatoid arthritis (27). We detected rituximab in urine from one patient with iMN 15 days after his first infusion.

We compared two regimens of rituximab in 2 cohorts of anti-PLA2R1 positive MN patients with similar clinical characteristics: 19 patients from Nice treated with two 1-g infusions at 2-week interval, and 27 patients from the GEMRITUX trial treated with two 375 mg/m$^2$ infusions at 1-week interval. We measured serum rituximab levels at months 3 and 6. Remissions occurred at month 6 in 13 (68.4 %) Nice patients and 8 (29.6%) GEMRITUX patients (p=0.02, 3 complete remissions vs none), and during follow-up in 16 (84.2%) Nice patients and 18 (66.6%) GEMRITUX patients (NS). Median time to remission was 3 and 9 months, respectively (p=0.02). The higher remission rate at month 6 was associated with higher serum rituximab levels at month 3 (p=0.004) and lower CD19 count at month 3 and month 6 (p=0.002 and 0.0004, respectively). In both treatment groups,

remission was associated with higher rituximab level (p=0.006), lower CD19 count (p=0.01) and PLA2R1 antibody titer (p=0.007) at month 3 (Seitz-Polski et al. in preparation). In conclusion, early clinical remission is dependent on rituximab protocol and correlated with serum rituximab level and CD19 count at month 3.

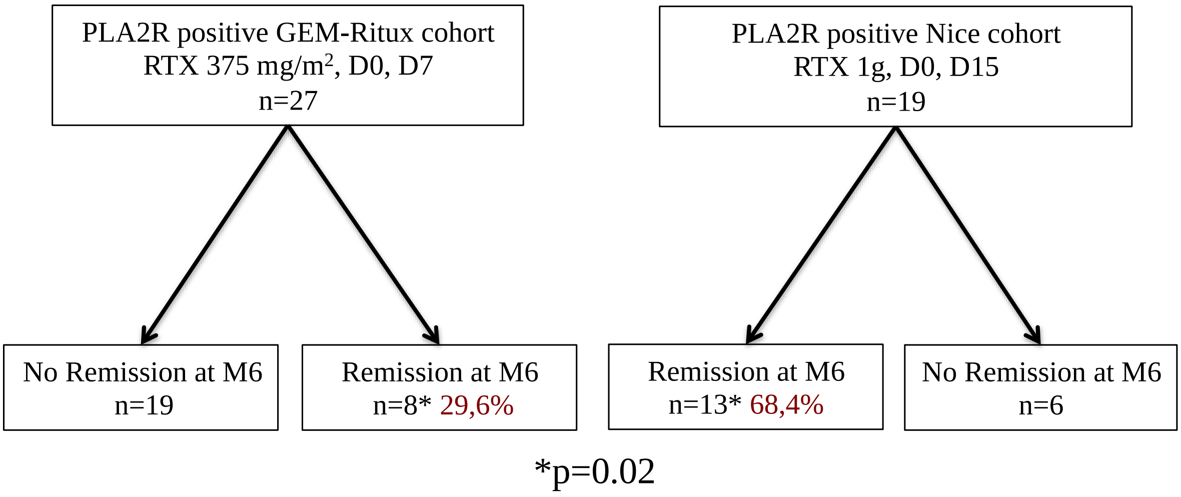


**Figure 4: Flowchart of the 19 patients from Nice and the 27 patients from GEMRITUX cohort, all with PLA2R1-Ab: outcome at month 6 after rituximab infusion**

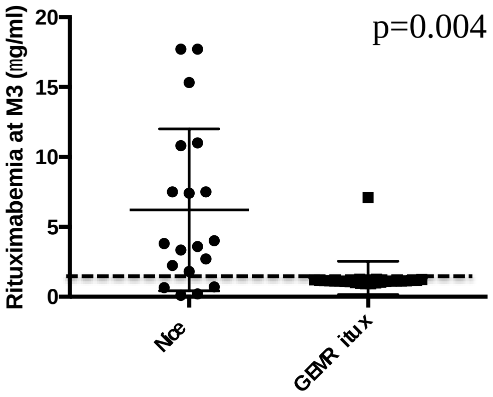


**Figure 5: Residual serum rituximab concentration at month 3.** Comparison of Nice (n=19, 2 infusions, 1g, 2-week interval) and GEMRITUX (n=27, 2 infusions, 375 mg/m$^2$, one-week interval) cohorts. Note the variability of residual rituximab concentrations and lower concentrations in the GEMRITUX patients.

In GEMRITUX cohort, remission rate at month 6 under rituximab was higher in patients with anti-PLA2R1 activity restricted to CysR than in patients with epitope spreading (70.0% vs 24.0% n=29 p=0.04).

***We concluded that:***

- ***early remission rate depends on rituximab dosing,***
- ***low dose of rituximab could be enough for patients with anti-PLA2R1 activity restricted to CysR,***
- ***high dose of rituximab may be required for patients with epitope spreading.***

We will compare two therapeutic strategies in PLA2R1-related iMN patients to induce clinical remission of the nephrotic syndrome at month 12:
1. The strategy validated in the GEMRITUX protocol: 6 months of symptomatic antihypertensive and antiproteinuric therapy, and if the nephrotic syndrome persists at month 6 (UPCR remains > 3.5 g/g and albuminemia < 30 g/l), two 375 mg/m$^2$ infusions at 1-week interval.

2. A personalized strategy driven by the PLA2R1-Ab epitope profile at month 0 and month 6:
- Patients with restricted anti-CysR activity at month 0: these patients have a spontaneous remission rate under NIAT from 43% to 50% at month-6. We propose a symptomatic antihypertensive and antiproteinuric treatment for 6 months;
- Patients with restricted anti-CysR activity and persisting active disease at month 6 after symptomatic treatment (UPCR remains > 3.5 g/g and albuminemia < 30 g/l): in GEMRITUX, these patients have a high rate of remission with low dose of rituximab (about 70%). We propose two 375 mg/m$^2$ rituximab infusions at 1-week interval;
- Patients with anti-CTLD1/7 activity at month 0 or month 6 with persisting active disease after symptomatic treatment (UPCR remains > 3.5 g/g and albuminemia < 30 g/l): these patients have a very low rate of spontaneous remission under NIAT (from 0.05% to 17%) or under low dose of rituximab: in GEMRITUX 24% of remission at 6-month vs 64% with high dose of rituximab p=0.04. We propose to treat these patients with high dose of rituximab (two 1g rituximab infusions at 2-week interval at month 0 and/or month 6).

Ruggenenti et al. demonstrated on a cohort of 81 consecutive iMN patients with PLA2R1-Ab that PLA2R1-Ab depletion 6 months post-rituximab strongly predicted remission (HR, 7.90; 95% CI, 2.54 to 24.60; P<0.001) (32). We will reevaluate the serologic statue of all patients at month-6 and retreat according to the same protocol.

Two studies showed that a significant increase in TReg cells from month 6 after rituximab infusion is associated with clinical response (33, 34). This increase is associated with IL-35 increase and PLA2R1-Ab decrease (33). IL-35 is an inhibitory cytokine that contribute to regulatory T-cell function. These results must be confirmed in large cohort to understand mechanisms of rituximab resistance and proposed new strategies (35). We propose to confirm these results in an ancillary study.

### 1.4 MN recurrences

The rate of iMN recurrences after rituximab infusion is about 30% (32). MN recurrences are associated with rise in PLA2R1-Ab titers (9, 32). In our cohorts (Nice cohort + GEMRITUX), 7 patients out of the 34 who reached remission relapsed. MN recurrences after rituximab were associated with the appearance of anti-rituximab antibodies at month 6 (p=0.02) and higher PLA2R1-Ab titer at month-3 and month-6 (p=0.04 and 0.03 respectively). In competition assays, anti-rituximab antibodies could block in vitro complement and cell dependent rituximab cytotoxicity on B-cells. Inhibition of rituximab activity could favor the persistence of pathogenic memory B-cells and induce disease relapse, with an early reconstitution of the B-cell compartment (36). We will monitor serum rituximab levels at month 3 and month-6, anti-rituximab antibodies at month-3 and month-6 as well as at inclusion for relapsing patients, and B-cell compartments (transitional, mature/naïve, memory) at Month 0, Month 3, Month 6, Month 12 and Month 18 after rituximab infusion to correlate the appearance of anti-rituximab antibodies and the re-emergence of the memory B-cell with MN recurrences in an ancillary study.

## 2. Study objectives

### 2.1 Primary objective

Our main objective is to compare the efficacy of a ***personalized treatment*** of nephrotic iMN driven by anti-PLA2R1 antibody epitope profile at month 0 and month 6 to induce clinical remission (partial or complete) of the nephrotic syndrome at month 12:

- Restricted anti-CysR activity at month 0: 6-month symptomatic treatment
- Restricted anti-CysR activity at month 6 after symptomatic treatment and if the nephrotic syndrome persists at month-6 (UPCR remains > 3.5 g/g and albuminemia < 30 g/l): two 375 mg/m$^2$ rituximab infusions at 1-week interval

- Anti-CTLD1/7 activity at month 0 or month 6: two 1g rituximab infusions at 2-week interval at month 0 and/or month 6

with the GEMRITUX protocol: 6 months of symptomatic antihypertensive and antiproteinuric therapy, and if the nephrotic syndrome persists at month-6 (UPCR remains > 3.5 g/g and albuminemia < 30 g/l), two 375 mg/$m^2$ infusions at 1-week interval. For patients who will develop anti-rituximab antibodies, treatment with human anti-CD20 is widely recommended.

### 2.2 Secondary Objectives

The following objectives are to compare between a personalized treatment of iMN driven by anti-PLA2R1 antibody epitope profiles and the GEMRITUX therapeutic protocol:

1. Complete clinical remissions at M6, M9, M12, M18, M24
2. Partial clinical remission at M6, M9, M12, M18, M24
3. Immunological remissions at M6, M9, M12, M18, M24
4. Proteinuria and albuminuria at M6, M9, M12, M18, M24
5. Changes in proteinuria and albuminuria from baseline at M6, M9, M12, M18, M24
6. Serum creatinine and estimated glomerular filtration rate (eGFR) at M6, M9, M12, M18
7. Changes in serum creatinine and eGFR from baseline at M6, M9, M12 and M18
8. PLA2R1-Ab titers at M6, M9, M12, M18 and M24
9. Recording all AEs/SAEs during study follow-up, 24 months

The following objectives aim to study risk factors for remission or relapse:

10. To determine risk factors for remission (immunological and clinical, partial or complete) at M12 in patients pooled from the two arms
11. To determine risk factors for relapse (between M12 and M24) in patient responders at M12, pooled from the two arms

## 3. Evaluation criteria

<u>Principal evaluation criteria:</u>

Clinical remission will be defined as a composite criterion combining (KDIGO definitions):

- Complete clinical remission: urinary protein/creatinine ratio (UPCR)<0.3 g/g in spot morning urine samples and Normal serum albumin and eGFR > 60 ml/min/1.73 $m^2$

- Partial clinical remission: UPCR < 3.5 g/g with a decrease greater than 50% from baseline and improvement or normalization of the serum albumin concentration and increase of serum creatinine lower than 20%

<u>Secondary evaluation criteria:</u>

1. Complete clinical remission as defined above
2. Partial clinical remission as defined above
3. Immunological remission: full PLA2R1 depletion measured by ELISA (titer<14RU/ml)
4. Proteinuria and albuminuria are measured on urine sample (proteinuria-to-creatinine ratio or albuminuria-to-creatinine ratio g/g) or collected during one day (g per day)
5. Change in proteinuria are measured in percentage change in proteinuria from baseline to M6, M9, M12, M18, M24
6. Serum creatinine is measured in blood sample in µmol/l and eGFR using the CKD-EPI formula.
7. Change in serum creatinine and eGFR are measured in percentage change from baseline to M6, M9, M12, M18, M24
8. PLA2R1-Ab titer are measured by ELISA in RU/ml
9. Severe infections are defined as infections that led to hospitalization during study follow-up
10. Remission will be defined as already stated. Patients from both groups will be analysed jointly. The following risk factors will be studied in this analysis:
    - Lymphocyte counts: B cells (CD19, transitional, mature and memory) and T cells (CD3, CD4, CD8 and TReg) measured in blood sample at J0, M3, M6, post-rituximab infusion
    - Serum level of cytokines (pg/ml) measured in blood sample at J0, and after first remission (or M24 if no remission)
    - Residual serum rituximab levels (µg/ml) measured in blood sample at M3 post-rituximab infusion using ELISA
    - Neutralizing anti-rituximab antibodies (ng/ml) measured in blood sample at M3 and M6 post-rituximab infusion
11. Responders are patients entered in partial or complete remission at M12. Relapse is defined by an increased proteinuria > 3.5 g/g after remission at M12. Patients from both groups will be analysed jointly. The risk factors studied will be the same as above measured at J0, M3, M6, M9 and M12 post-rituximab infusion.

## 4. Type of study

Randomized, open label, multicenter (23 sites), prospective trial comparing the efficacy of two strategies to obtain a clinical remission of the nephrotic syndrome 1 year after diagnosis of iMN with anti-PLA2R1 antibodies. As this project is research with category 1 involvement of human beings, it is subject to the provisions of the French Public Health (Law n°2012-300 of 5 March 2012) regarding research involving human beings, as altered by decree n°2016-800 of 16 June 2016.
The study will last 6 years: inclusion period will last 4 years with 2 years of follow-up.

## 5. Benefit/ Risk

<u>Benefit</u>

- At the individual level:

Our recent works on the identification of nephrotoxic autoantibody activity should contribute to improve the individual management of patients with MN, and help to better select patients who should benefit from early and aggressive immunosuppressive strategies. Patients in the personalized arm should expect a remission rate around 85% at 12 months.

- At the collective level:

If the personalized strategy is validated, we should improve the remission rate and decrease the risk of serious adverse events in iMN treatment. Moreover, ancillary studies will help us to understand resistant forms of iMN and proposed new immune therapy like inducing TReg therapy, or Memory B-cell targeting in recurrent forms.

<u>Risk</u>

With a dose of 375 $mg/mm^2$, the most frequently observed adverse drug reactions (ADRs) in patients receiving rituximab were infusions related reactions (IRRs) which occurred in the majority of patients during the first infusion. The incidence of infusion-related symptoms decreases substantially with subsequent infusions and is less than 1% after eight doses of rituximab.
Infectious events (predominantly bacterial and viral) occurred in approximately 30-55% of patients during clinical trials in patients with NHL and in 30-50% of patients during clinical trials in patients with CLL

The most frequent reported or observed serious adverse drug reactions were:

- IRRs (including cytokine-release syndrome, tumor-lysis syndrome), see page 34
- Infections, see page 34
- Cardiovascular events, see page 34

Other serious ADRs reported include hepatitis B reactivation and multifocal leukoencephalopathy (page34)

With a dose of 1g/mm2, the most frequent adverse reactions considered due to receipt of rituximab were IRRs. The overall incidence of IRRs in clinical trials was 23% with the first infusion and decreased with subsequent infusions. Serious IRRs were uncommon (0.5% of patients) and were predominantly seen during the initial course. In addition to adverse reactions seen in RA clinical trials for rituximab, progressive multifocal leukoencephalopathy (PML) (page 34) and serum sickness-like reaction have been reported during post marketing experience.
Our protocol does not increase the number of visits of a conventional follow-up.

Conclusion: All things considered, the risk/benefit balance appears to be favorable in this study.

## 6. Selection and exclusion criteria for study participants

### 6.1 Inclusion criteria

- Age 18 years or more
- Anti-PLA2R1 activity detected by ELISA or Euroimmune IFA
- Nephrotic syndrome defined by proteinuria > 3.5 g/24h (or UPCR > 3.5 g/g) and serum albumin < 30 g/L at diagnosis
- eGFR (CKD-EPI) > 30 ml/min/1,73 $m^2$ at diagnosis
- Symptomatic treatment according to KDIGO guidelines: maximal tolerated dose of NIAT (angiotensin-converting enzyme inhibitor and/or angiotensin 2 receptor blockers, diuretics and statins)
- Medical insurance
- Signed informed consent
- Having understood and accepted the need for long-term medical follow-up
- Woman of child-bearing age must be using an effective method of contraception

## 6.2 Exclusion criteria

- Secondary MN: MN related to cancer, infectious, systemic lupus erythematosis, drug
- Anti-PLA2R1 antibodies not confirmed by central analysis (in this case the patient will be replaced)
- Pregnancy or breastfeeding
- Immunosuppressive treatment in the 6 last months
- Presence of anti-rituximab antibodies for relapsed patients
- Cancer under treatment
- Patient with complicated nephrotic syndrome that would require early immunosuppressive treatment (thrombosis, acute renal failure…)
- Patients with active, severe infections or active hepatitis B
- Hypersensitivity to the active substance or to murine proteins, or to any of the other excipients
- Patients in a severely immunocompromised state
- Severe heart failure (New York Heart Association Class IV) or severe, uncontrolled cardiac disease
- Patients unable to give an informed consent

# 7. Sample size calculation

## 7.1 Estimate of remission rate in the personalized arm

According to our preliminary data, about 30% of patient with iMN have CysR restricted activity at diagnosis. About 50% will enter in spontaneous remission after 6 months of NIAT and 40% after NIAT + Rituximab at low doses (375 mg/m$^2$ D0 and D7).

About 70% of patients have CTLD1/7 activity, in our cohorts, about 85% entered in remission after repeated pulses of high doses of rituximab.

We therefore expect a remission rate of 80% at M12 in the personalized arm.

## 7.2 Estimate of remission rate in the control arm

Based on data from the literature 10-21% of iMN patient with nephrotic syndrome entered into spontaneous remission after 1-year of NIAT (11, 18) and 35% in GEMRITUX after NIAT + low doses of rituximab (375 mg/m$^2$ D0 and D7) (18).

We expect a remission rate of 45% in the control arm.

With α=0,20 and β=0.05 (two-sided test), the number of patients required is 29 in each group (Nquery© Advisor v 7.0, two group Fisher's-exact test). To account for a 10% rate of lost to follow-up (anticipated as minor in this study as patients are intensively followed for this pathology), the global sample size is 64 patients.
This sample size is large in the frame of this study because the medical condition studied here is rare. Actually, we can expect an annual eligible number of patients of 1 or 2 in the 23 centers, which are in charge of almost all the cases nationally (reference centers). Since 50% of patients may refuse to participate, we estimate that 4 years are required to recruit the calculated sample size.

## 8. Description of the conduct of the study

### 8.1 Conventional management of iMN patient

All adult patients with a nephrotic syndrome should receive symptomatic treatment. When the diagnosis of MN is confirmed by anatomopathology and/or serology (positive Anti-PLA2R antibodies), additional tests comprise scanner TAP, serologies (HBV, HBC), anti-nuclear and anti THSD7A antibodies. After 6 months of NIAT and a persistent nephrotic syndrome, an immunosuppressive therapy is started. They have a follow-up every 3 months.

### 8.2 Patient selection for this study

Patients with clinical diagnosis of MN will be tested for the presence of PLA2R1-Ab according to local practices. The selection of the patients fulfilling the inclusion criteria will not delay the start of the symptomatic treatment according to KDIGO guidelines. If the patient meets the criteria for recruitment to the study, they will be informed of the study by the referring nephrologist. Full information will be supplied orally, together with written information.

### 8.3 Inclusion and randomization = M0

After signing the study consent, participants will be randomized in one of the two treatment arms:

- GEMRITUX treatment
- Personalized treatment

Randomization will be balanced (1:1). Centralized block randomization will be conducted by the *Département de la Recherche Clinique et de l'Innovation* (DRCI) (Department of Clinical Research and Innovation) at CHU Nice (Nice University Hospital). Taking into account the size of our study

sample which is relatively small and the high number of participating centers, (which is high in light of the need to ensure that the intended level of recruitment is met), it does not seem appropriate to account for the center as a stratification parameter.

Randomization will be integrated in the electronic case report file (e-CRF) devised specifically for the study with Redcap® software. Using their personal access details to login, the investigator will provide the necessary patient information (i.e. the first letter of their first and last name and their month and year of birth) for random allocation to treatment by the online randomization module (Redcap®). Randomization can then take place around the clock. The treatment group and inclusion number for the patient will then be relayed to the investigator and the central lab team. The patient's trial records will then be created automatically, allowing data to be entered.

### 8.4 Frequency of follow-up visit-protocol

After randomization, all patients will be scheduled at M3, M6, M9, M12, M18 and M24 post-treatment for a routine follow-up, and benefit from a symptomatic antihypertensive and antiproteinuric treatment throughout the study period according to the KDIGO guidelines.

In the GEMRITUX group: at M6, if the patient exhibits an active disease (UPCR remains > 3.5 g/g and albuminemia < 30 g/l), they will receive two 375 mg/$m^2$ rituximab infusions at 1-week interval.

In the personalized group, rituximab injections should be scheduled between 5 and 14 days after randomization in case the epitope results are in favor for immunosuppressive treatment, as follows:

- at M0 and M6, if the patient exhibits anti-PLA2R1 anti-CTLD1 or CTLD7 activity, they will receive two 1 g infusions at 2-week interval.
- At M6, if a patient has an active disease/ anti-PLA2R1 ab + or if he has an active disease /anti-PLA2R1 Ab –, symptomatic treatment should be optimized. And if he exhibits anti-CysR restricted activity with active disease (UPCR remains > 3.5 g/g and albuminemia < 30 g/l), they will receive two 375-mg/$m^2$ rituximab infusions at 1-week interval.

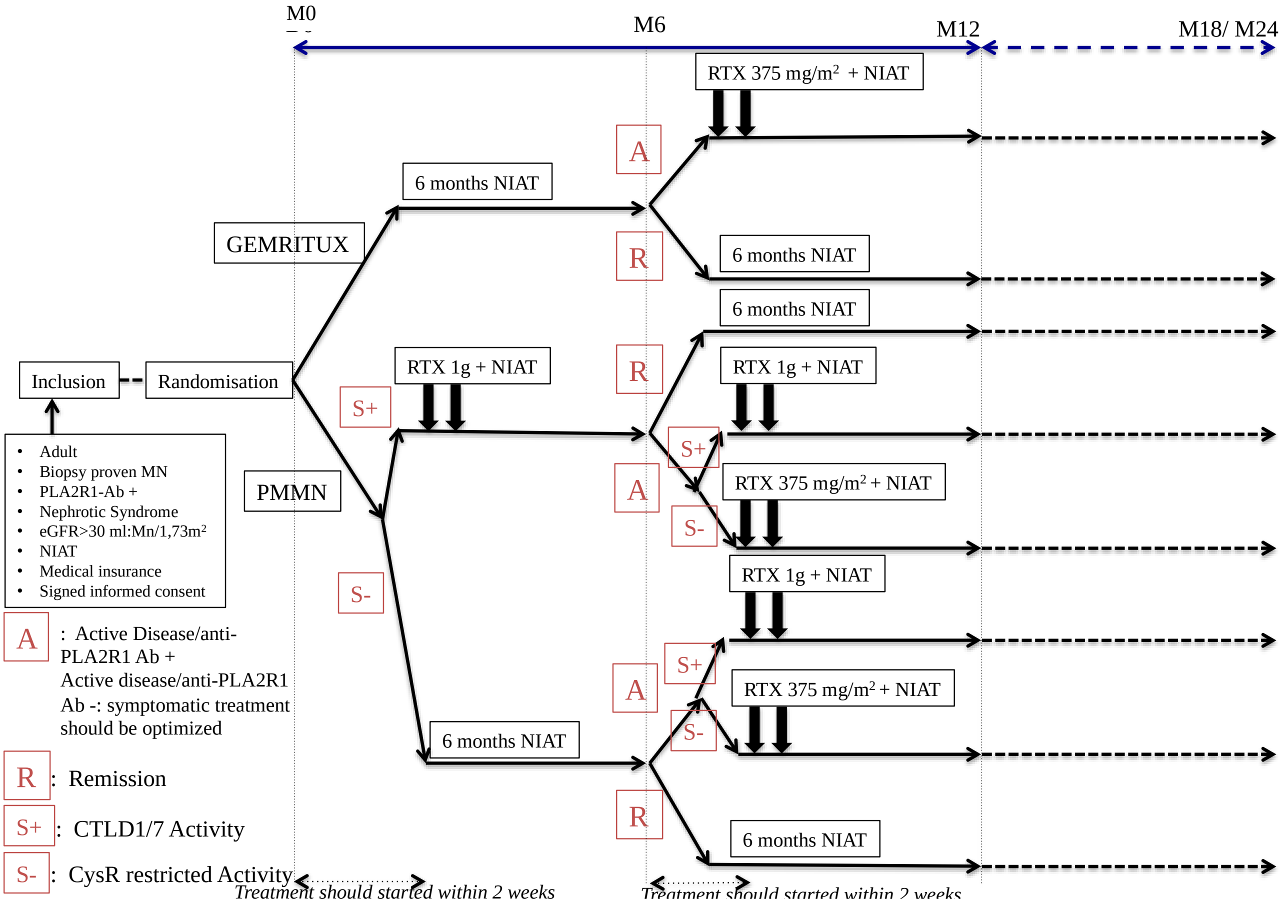


## 8.5 Definition of patient data to be gathered and directly entered in the patient electronic case file, which will be considered the source data:

### Inclusion/Randomization visit (M0)

- Consultation date
- Demographic data: date of birth, sex
- History of MN
- Paraclinic tests to eliminate secondary MN when performed: body scan, anti-nuclear antibodies, HBV, HCV serologies
- Current treatment
- Clinical examination: weight, height, blood pressure, edema
- Blood sample: urea, creatinine, electrolytes (Na, K, Cl, $HCO_3$), protein, albumin, full blood count
- Urine collection on a spot morning sample: protein, albumin, urea, creatinine
- Centralized assays : for both groups,

  - 2 dry tubes of 5 ml of blood and 2 EDTA tubes of 3 ml of blood will be shipped to Nice at room temperature for PLA2R1-Ab, epitope profile determination, anti-rituximab antibodies, B and T cells counts (see manual lab),
  - 1 lithium heparin tube of 4 ml of blood for cytokine profile analysis will be shipped to Nice at 4°C.

- Start vaccination (pneumococcal and influenza vaccine) between randomization and first injection at the latest.

- <u>In the GEMRITUX arm,</u> plan M3 in 3 months +/-15days and hand out a prescription to the patient for blood and urine analyses* (results needed for the next visit as per routine follow-up);
- <u>In the Personalized Treatment arm</u>, plan next visit according to the epitope profile results provided by the central lab within 48 hours:
  - Restricted anti-CysR activity: 6-month symptomatic treatment as in GEMRITUX arm -> plan M3 visit in 3 months +/-15 days and hand out a prescription to the patient for blood and urine analyses* (results needed for the next visit as per routine follow-up);
  - Anti-CTLD1/7 activity: plan two 1g rituximab infusions at 2-week interval +/-2 days. The treatment should start between 5 and 14 days after randomization. After the first injection, plan M3 visit in 3 months +/-15 days and hand out a prescription to the patient for blood and urine analyses* (results needed for the next visit as per routine follow-up)

* Blood sample: urea, creatinine, electrolytes (sodium, potassium, chloride, bicarbonate), protein, albumin, full blood count.
Spot morning urine sample: protein, albumin, urea, creatinine.

**M3 visit = 3 months after +/-15 days**

- Consultation date
- Clinical examination: weight, blood pressure, edema
- Serious adverse events
- Changes in concomitant treatments
- Blood and urine lab results

- Centralized assays for both arms:
    - 2 dry tubes of 5 ml of blood and 2 EDTA tubes of 3 ml of blood will be sent to Nice at room temperature for PLA2R1-Ab, epitope profile determination, rituximab serum levels, anti-rituximab antibodies, and B and T cells counts (see manual lab)
- Plan M6 visit +/-15 days and hand out a prescription to the patient for blood and urine analyses*

* Blood sample: urea, creatinine, electrolytes (sodium, potassium, chloride, bicarbonate), protein, albumin, full blood count.

Spot morning urine sample: protein, albumin, urea, creatinine.

**M6 visit = 6 months after randomization +/-15 days**

- Consultation date
- Clinical examination: weight, blood pressure, edema
- Serious adverse events
- Changes in concomitant treatments
- Blood and urine lab results
- Estimated GFR CKD EPI
- Centralized assays for both arms:
    - 2 dry tubes of 5 ml of blood and 2 EDTA tubes of 3 ml of blood will be shipped to Nice at room temperature for PLA2R1-Ab, epitope profile determination, serum rituximab levels, anti-rituximab antibodies, and, B and T cells counts (see manual lab)

- **<u>In the GEMRITUX arm</u>**
    - If disease still active (UPCR remains > 3.5 g/g and albuminemia < 30 g/l): plan two 375 mg/m² Rituximab infusions at 1-week interval (+/- 2 days) between 5 and 14 days after M6 visit.
    - If remission (partial or complete, see p.12): symptomatic treatment to be continued.

- **<u>In the personalized arm/ Patients who received RTX at M0</u>**
    - If remission (partial or complete, see p.12): symptomatic treatment to be continued alone and contraception will be required during 12 months after the end of the treatment.
    - If disease still active / Restricted anti-CysR activity: plan two 375 mg/m$^2$ rituximab infusions at 1-week interval (+/- 2 days) between 5 and 14 days after M6 visit.

- if disease still active / Anti-CTLD1/7 activity: plan two 1g rituximab infusions at 2-week interval (+/- 2 days) between 5 and 14 days after M6 visit. For patient who will develop anti-rituximab antibodies, treatment with human anti-CD20 is widely recommended.

*NB: If the patient still active disease / anti-PLA2R1 Ab-; the symptomatic treatment should be optimized*

- **<u>In the personalized arm/ Patients who received 6-month symptomatic treatment</u>**
  - If remission (partial or complete, see p.12): symptomatic treatment to be continued
  - If disease still active / Restricted anti-CysR activity: plan two 375 mg/m$^2$ rituximab infusions at 1-week interval between 5 and 14 days after M6 visit.
  - if disease still active / Anti-CTLD1/7 activity: plan two 1g rituximab infusions at 2-week interval between 5 and 14 days after M6 visit.

**For all**: plan M9 +/-15 days and hand out a prescription to the patient for blood and urine analyses*

* Blood sample: urea, creatinine, electrolytes (sodium, potassium, chloride, bicarbonate), protein, albumin, full blood count.
Spot morning urine sample: protein, albumin, urea, creatinine.

**M9 visit = 9 months after randomization +/-15 days**

- Consultation date
- Clinical examination: weight, blood pressure, edema
- Serious adverse events
- Changes in concomitant treatments
- Blood and urine lab results
- Estimated GFR CKD EPI
- Check remission status:
  - Complete clinical remission: urinary protein/creatinine ratio (UPCR) < 0.3 g/g in spot morning urine samples and Normal serum albuminand eGFR > 60 ml/min/1.73 m2
  - Partial clinical remission: UPCR < 3.5 g/g with a decrease greater than 50% from baseline and improvement or normalization of the serum albumin concentrationand increase of serum creatinine lower than 20%
- Centralized assays for both arms:

    - 2 dry tubes of 5 ml of blood and 2 EDTA tubes of 3 ml of blood will be sent to Nice at room temperature for PLA2R1-Ab, epitope profile determination, serum rituximab levels, anti-rituximab antibodies, and B and T cells counts (see manual lab)

- Plan visit M12 +/-15 days and hand out a prescription to the patient for blood and urine analyses*

* Blood sample: urea, creatinine, electrolytes (sodium, potassium, chloride, bicarbonate), protein, albumin, full blood count.
Spot morning urine sample: protein, albumin, urea, creatinine.

**M12 visit = 12 months after randomization +/-15 days**

- Consultation date
- Clinical examination: weight, blood pressure, edema
- Serious adverse events
- Changes in concomitant treatments
- Blood and urine lab results
- Estimated GFR CKD EPI
- Check remission status:
  - Complete clinical remission: urinary protein/creatinine ratio (UPCR) < 0.3 g/g in spot morning urine samples and Normal serum albuminand eGFR > 60 ml/min/1.73 m2
  - Partial clinical remission: UPCR < 3.5 g/g with a decrease greater than 50% from baseline and improvement or normalization of the serum albumin concentrationand increase of serum creatinine lower than 20%
- Centralized assays for both arms:
    - 2 dry tubes of 5 ml of blood and 2 EDTA tubes of 3 ml of blood will be shipped to Nice at room temperature for PLA2R1-Ab, epitope profile determination, serum rituximab levels, anti-rituximab antibodies, B and T cells counts (see manual lab)
- Plan visit M18 +/-15 days and hand out a prescription to the patient for blood and urine analyses*
- For all patients treated by rituximab, contraception of 12 months will be requiring after the end of the treatment.

* Blood sample: urea, creatinine, electrolytes (sodium, potassium, chloride, bicarbonate), protein, albumin, full blood count.
Spot morning urine sample: protein, albumin, urea, creatinine.

**M18 visit = 18 months after randomization +/-15 days**

- Consultation date
- Clinical examination: weight, blood pressure, edema
- Serious adverse events
- Changes in concomitant treatments
- Blood and urine lab results
- Estimated GFR CKD EPI
- Check remission status:
  - Complete clinical remission: urinary protein/creatinine ratio (UPCR)<0.3 g/g in spot morning urine samples and Normal serum albumin and eGFR > 60 ml/min/1.73 m2
  - Partial clinical remission: UPCR < 3.5 g/g with a decrease greater than 50% from baseline and improvement or normalization of the serum albumin concentration and increase of serum creatinine lower than 20%
- Centralized assays for both arms:
  - 2 dry tubes of 5 ml of blood and 2 EDTA tubes of 3 ml of blood will be shipped to Nice at room temperature for PLA2R1-Ab, epitope profile determination, B and T cells counts (see manual labPlan visit M24+/-15 days and hand out a prescription to the patient for blood and urine analyses*

*Blood sample: urea, creatinine, electrolytes (sodium, potassium, chloride, bicarbonate), protein, albumin, full blood count.
Spot morning urine sample: protein, albumin, urea, creatinine.

**M24 visit = 24 months after randomization +/-15 days**

- Consultation date
- Clinical examination: weight, blood pressure, edema
- Serious adverse events
- Changes in concomitant treatments
- Blood and urine lab results

- Estimated GFR CKD EPI
- Check remission status:
    - Complete clinical remission: urinary protein/creatinine ratio (UPCR)<0.3 g/g in spot morning urine samples and Normal serum albumin and eGFR > 60 ml/min/1.73 m2
    - Partial clinical remission: UPCR < 3.5 g/g with a decrease greater than 50% from baseline and improvement or normalization of the serum albumin concentration and increase of serum creatinine lower than 20%
- Centralized assays for both arms:
    - 2 dry tubes of 5 ml of blood and 2 EDTA tubes of 3 ml of blood will be shipped to Nice at room temperature for PLA2R1-Ab, epitope profile determination, B and T cells counts (see manual lab)
- End of study

**At the first remission**

One tube of lithium heparin of 4 ml of blood (for cytokine profile analysis) will be performed at the first remission and shipped to Nice at 4°C. If the sample cannot be collected during the visit when the remission as first confirmed, it must be collected at the following visit or at M24 for all patients that have not entered remission until the end of study.

**At the first relapse**

If the patient presents a still active disease with <u>anti-PLA2R1 and anti-rituximab activity</u>, the following cares are left to the investigator's discretion due to the Rituximab contraindication. However, the patient must perform the following visits according to the protocol.

**8.7 Specimen collection:**

Non-fasted blood samples will be shipped on the same day at room temperature to the Laboratory of Immunology in Nice, overseen by Dr Seitz-Polski (see instructions in manual lab), except for the tubes of lithium heparin (collected at inclusion and at first remission) that must be immediately stored at 4°C and shipped at 4°C. They will be analyzed and results will be provided to the study site within 48 hours. All samples will be retained after analysis (except if consent for this is withdrawn) to pursue other research goals (they will constitute a collection) and will be stored at -80°C in a secured fridge/freezer located in the Laboratory of Immunology in Nice Hospital. No genetic study

will be performed on the samples. The Nice Universtiy Hospital where the collection is stored will be responsible for declaring it to the Research minister and the Agence Régionale d'Hospitalisation. The cell bank has all of the necessary informatic tools and software necessary for conformance with the national bioethical legislation (loi de Bioéthique). The rules governing sample anonymity will be followed.

**Schedule of assessments**

| | M0 | M3 | M6 | M9 | M12 | M18 | M24 |
|---|---|---|---|---|---|---|---|
| Selection criteria | X | | | | | | |
| Informed Consent | X | | | | | | |
| Pregnancy test | X | X | X | X | X | | |
| Randomisation | X | | | | | | |
| Medical history and demographics | X | | | | | | |
| Weight, Edema, Blood pressure | X | X | X | X | X | X | X |
| Vaccination | X | | | | | | |
| HBP Medications | X | X | X | X | X | X | X |
| Serious adverse events treatment related | X | X | X | X | X | X | X |
| Estimated GFR CKD EPI | | | X | X | X | X | X |
| Urine analyses | X | X | X | X | X | X | X |
| Blood sample[b] | X | X | X | X | X | X | X |
| PLA2R1-Ab | X | X | X | X | X | X | X |
| Epitope Profile | X | X | X | X | X | X | X |
| Residual Rituximabemia | | X | X | X | X | | |
| Rituximab-Ab | X | X | X | X | X | | |
| Cytokine profile | X | X At first remission [a] | | | | | |
| B and T cells count | X | X | X | X | X | X | X |
| End of study | | | | | | | X |

*[a]:If the sample cannot be collected at the visit when the remission is confirmed, it must be collected at the following visit.*
*[b]: urea, creatinine, electrolytes (sodium, potassium, chloride, bicarbonate), protein, albumin, full blood count.*

## 9. Treatment

### 9.1 Study treatment

**- Investigational Product**

The investigational product rituximab will be stored in a secure area according to local regulations. It will be the responsibility of the investigator to ensure that investigational product is only

dispensed to study subjects. The investigational product will be dispensed only from official study sites by authorized personnel according to local regulations.
Rituximab will be delivered at two different doses according to the arm of the protocol: 1g J0 and J15 in the personalized arm with anti-CTLD1/7 activity or 375 mg/$m^2$ J0 and J7 in the personalized arm with anti-CysR activity and active disease at M6 or in the GEMRITUX arm with active disease.

**- Packaging and Labeling**

Celltrion® / Biogaran® will provide Rituximab (Truxima®) at no cost for this study. Rituximab will be provided in open-label containers. The labels will contain the protocol prefix, batch number, content, storage conditions, and dispensing instructions along with the Investigational New Drug (IND) caution statement.

**- Handling and Dispensing**

The product storage manager will ensure that the study drug is stored in accordance with the environmental conditions (temperature, light, and humidity) as determined by Celltrion®/ Biogaran ®. If concerns regarding the quality or appearance of the study drug arise, the study drug will not be dispensed and Celltrion®/ Biogaran® will be contacted immediately.
Investigational product documentation will be maintained and include all processes required to ensure drug is accurately administered. This includes documentation of drug storage, administration and, as applicable, storage temperatures, reconstitution, and use of required processes (e.g., required diluents, administration sets).
Investigation/non-investigational products that are procured as local marketed product should be stored in accordance with the package insert, summary of product characteristics (SmPC) or equivalent document.
Rituximab vials will be stored at a temperature of 2°C to 8°C and will be protected from light. If stored in a glass front refrigerator, vials will be stored in the carton. Recommended safety measures for preparation and handling of rituximab will include laboratory coats and gloves.
The investigational sites are responsible for providing IV bags, diluents, filters etc.
For details on prepared drug storage and use time of Rituximab under room temperature/light and refrigeration, please refer to the Investigator Brochure section for “Recommended Storage and Use Conditions.”
Care will be taken to assure sterility of the prepared solution as the product does not contain any anti-microbial preservative or bacteriostatic agent.

Rituximab will be delivered after the first randomization in the center if the patient is in PMMN arm.
2 treatments of Rituximab will be sent between 2°C and 8°C with specific transporter.
Rituximab will be to be administered IV infusion, using a volumetric pump start at 100 mg/h then increase to 400 mg/h. The drug can be diluted with 0.9% normal saline or glucosal 5% for delivery but the total drug concentration of the solution cannot be below 1 mg/ml. It is not to be administered as an IV push or bolus injection. At the end of the infusion, flush the line with a sufficient quantity of normal saline.
Rituximab could be associated with perfalgan 1g IV, Polaramine 5 mg and Solumedrol 100 mg.

### 9.2 Medical treatment common to both arms of the study: Nephro-protective treatment

The KDIGO 2012 recommendations for the management of nephrotic syndrome will be followed:
First line RAAS blockade with an angiotensin-converting enzyme inhibitor (ACEI) or an angiotensin 2 receptor antagonist and diuretic up-titration.
Double RAAS blockade could be indicated under close monitoring.
Drugs employed for the symptomatic treatment are those uses in usual practice. Each center will be referring to the internet website: http://base-donnees-publique.medicaments for adapting the contraindications, contraception, monitoring of patients and prohibit drugs in accordance with the study (annexe 1).

### 9.3 Concomitant Treatments

**- Prohibited and/or Restricted Treatments**

Immunosuppressive agents or immunosuppressive doses of systemic corticosteroids are prohibited during the study (unless utilized to treat a drug related adverse event).

**- Permitted therapy**

Subjects are permitted the use of topical, ocular, intra-articular, intranasal and inhalational corticosteroids (with minimal systemic absorption). Physiologic replacement doses of systemic corticosteroids are permitted even if > 10 mg daily prednisone (or equivalent). A brief course of corticosteroids for the prevention (e.g., for contrast dye allergy) or treatment of non-autoimmune conditions (e.g., delayed-type hypersensitivity reaction caused by a contact allergen) is permitted.
Concomitant medications are recorded at screening/baseline and throughout the treatment phase of the study, in the appropriate section of the CRF. All medications (prescriptions or over the counter

medications) continued at the start of the study or started during the study and different from the study drug must be documented in the concomitant therapy section of the CRF.

## 10. Reporting adverse events (AEs) and serious adverse events (SAEs)

### 10.1 Definitions

| | |
|---|---|
| Adverse Event (AE) | Any untoward medical occurrence in a participant to whom a medicinal product has been administered, including occurrences that are not necessarily caused by or related to that product. |
| Adverse Reaction (AR) | An untoward and unintended response in a participant to an investigational medicinal product, which is related to, any dose administered to that participant.<br><br>The phrase "response to an investigational medicinal product" means that a causal relationship between a trial medication and an AE is at least a reasonable possibility, i.e. the relationship cannot be ruled out.<br><br>All cases judged by either the reporting medically qualified professional or the Sponsor as having a reasonable suspected causal relationship to the trial medication qualify as adverse reactions. |
| Serious Adverse Event (SAE) | A serious adverse event is any untoward medical occurrence that:<br><br>Results in death<br><br>Is life-threatening<br><br>Requires patient hospitalisation or prolongation of existing hospitalisation<br><br>Results in persistent or significant disability/incapacity<br><br>Consists of a congenital anomaly or birth defect. |

| | |
|---|---|
| | Other 'important medical events' may also be considered serious if they jeopardise the participant or require an intervention to prevent one of the above consequences.<br><br>NOTE: The term "life-threatening" in the definition of "serious" refers to an event in which the participant was at risk of death at the time of the event; it does not refer to an event, which hypothetically might have caused death if it were more severe. |
| Serious Adverse Reaction (SAR) | An adverse event that is both serious and, in the opinion of the reporting Investigator, believed with reasonable probability to be due to one of the trial treatments, based on the information provided. |
| Suspected Unexpected Serious Adverse Reaction (SUSAR) | A serious adverse reaction, the nature and severity of which is not consistent with the information about the medicinal product in question set out:<br><br>In the case of a product with a marketing authorisation, in the summary of product characteristics (SmPC) for that product<br><br>In the case of any other investigational medicinal product, in the investigator's brochure (IB) relating to the trial in question. |

NB: to avoid confusion or misunderstanding of the difference between the terms "serious" and "severe", the following note of clarification is provided: "Severe" is often used to describe intensity of a specific event, which may be of relatively minor medical significance. "Seriousness" is the regulatory definition supplied above.

### 10.2 Causality

The relationship of each adverse event to the trial medication must be determined by a medically qualified individual (the Country CI or delegate) according to the following definitions:

- reasonable possibility
- no reasonable possibility

All AEs (SAEs) labelled reasonable possibility of a causal relationship to the IMP will be considered as related to the IMP.

### 10.3 Adverse Event Reporting

An adverse event is any condition that occurs after enrollment, whether or not considered related to study treatment. Signs and symptoms existing prior to study and documented on the baseline Medical History form or Physical Examination Form are not considered AEs. Only baseline signs and symptoms that worsen while the subject is on the study drug are considered adverse events.

At every visit, participant will be questioned regarding the occurrence and nature of any adverse experiences. If any signs, symptoms or the results of the laboratory tests indicate an adverse effect, the study physician should determine the severity (grade) of the AE and judge the relationship to the study treatment. In addition, if an adverse event necessitates medical care, appropriate care will be provided. Adverse events should be documented on the Adverse Event Report form, regardless of relationship to the study drugs. Notably, AEs thought to be due to treatment with the study drug are still reported as AEs in this study. The following information will be collected for all Adverse Events:

- Start and stop dates
- Severity (See the Severity Grading section below)
- Relationship to study drugs (See the Association with the Study Drug below )
- Action taken with study drugs
- Treatment for the AE and outcomes

The only circumstance in which an Adverse Event Report Form will not be filled out will be isolated lab value abnormalities of grade 1 severity.

<u>Severity Grading</u>

For evaluation and reporting purposes, events will be graded by using CTCAE v5.0.:

Grade refers to the severity of the AE. The CTCAE displays Grades 1 through 5 with unique clinical descriptions of severity for each AE based on this general guideline:

- **Grade 1** Mild; asymptomatic or mild symptoms; clinical or diagnostic observations only; intervention not indicated.

- **Grade 2** Moderate; minimal, local or noninvasive intervention indicated; limiting age-appropriate instrumental ADL*.
- **Grade 3** Severe or medically significant but not immediately life-threatening; hospitalization or prolongation of hospitalization indicated; disabling; limiting self care ADL**.
- **Grade 4** Life-threatening consequences; urgent intervention indicated.
- **Grade 5** Death related to AE.

**Activities of Daily Living (ADL)**
*Instrumental ADL refer to preparing meals, shopping for groceries or clothes, using the telephone, managing money, etc.
**Self care ADL refer to bathing, dressing and undressing, feeding self, using the toilet, taking medications, and not bedridden.

### 10.4 Association with the Study Drug

- Association with the use of the study drug means that there is a reasonable possibility that the adverse event may have been caused by the drug under investigation. All adverse events are graded with regard to their association with the use of the study drug. The classifications used includes reasonable possibility or no reasonable possibility of a causal relationship to the IMP.

### 10.5 SAE and SUSAR Reporting Procedure

**Electronic database for safety reporting**

1. The European Medicines Agency established by Regulation (EC) No 726/2004 (the 'Agency') shall set up and maintain an electronic database for the reporting.
2. The Agency shall, in collaboration with Member States, develop a standard web-based structured form for the reporting by sponsors to the database referred to in paragraph 1 of suspected unexpected serious adverse reactions.

**Reporting of adverse events and serious adverse events by the investigator to the sponsor**

The investigator shall record and document adverse events or laboratory abnormalities identified in the protocol as critical to the safety evaluation and report them to the sponsor in accordance with the reporting requirements and within the periods specified in the protocol.

The investigator shall record and document all adverse events, unless the protocol provides differently. The investigator shall report to the sponsor all serious adverse events occurring to subjects treated by him or her in the clinical trial, unless the protocol provides differently.

The investigator shall report serious adverse events to the sponsor **without undue delay** but not later than within 24 hours of obtaining knowledge of the events, unless, for certain serious adverse events, the protocol provides that no immediate reporting is required. Where relevant, the investigator shall send a follow-up report to the sponsor to allow the sponsor to assess whether the serious adverse event has an impact on the benefit-risk balance of the clinical trial.

The sponsor shall keep detailed records of all adverse events reported to it by the investigator.

If the investigator becomes aware of a serious adverse event with a suspected causal relationship to the investigational medicinal product that occurs after the end of the clinical trial in a subject treated by him or her, the investigator shall, without undue delay, report the serious adverse event to the sponsor.

All serious adverse events (SAE) must be reported by the investigator to the sponsor’s service in charge of safety (Vigilance):
Clinical Trials Vigilance: Olga KROSELJ, CHU de Nice; Tel: 0492034636; Fax: 0492037513;
email: drci-vigilance-ec@chu-nice.fr

## 10.6 Reporting of suspected unexpected serious adverse reactions by the sponsor to the Agency

1. The sponsor performed in at least one Member State shall report electronically and without delay to the database all relevant information about the following suspected unexpected serious adverse reactions:
(a) all suspected unexpected serious adverse reactions to investigational medicinal products occurring in that clinical trial, irrespective of whether the suspected unexpected serious adverse reaction has occurred at a clinical trial site in the Union or in a third country;

(b) all suspected unexpected serious adverse reactions to investigational medicinal products occurring in any of the subjects of the clinical trial, which are identified by or come to the attention of the sponsor after the end of the clinical trial.

2. The period for the reporting of suspected unexpected serious adverse reactions by the sponsor to the Agency shall take account of the seriousness of the reaction and shall be as follows:

(a) in the case of fatal or life-threatening suspected unexpected serious adverse reactions, as soon as possible and in any event not later than seven days after the sponsor became aware of the reaction;
(b) in the case of non-fatal or non-life-threatening suspected unexpected serious adverse reactions, not later than 15 days after the sponsor became aware of the reaction;
(c) in the case of a suspected unexpected serious adverse reaction which was initially considered to be non-fatal or non- life threatening but which turns out to be fatal or life-threatening, as soon as possible and in any event not later than seven days after the sponsor became aware of the reaction being fatal or life-threatening.

Where necessary to ensure timely reporting, the sponsor may submit an initial incomplete report followed up by a complete report.

3. Where a sponsor, due to a lack of resources, does not have the possibility to report to the database referred to in Article 10.5.1 and the sponsor has the agreement of the Member State concerned, it may report to the Member State where the suspected unexpected serious adverse reaction occurred. That Member State shall report the suspected unexpected serious adverse reaction.

### 10.7 Exception rules for SAE reporting

Exceptions to immediate SAE reporting are applied if hospitalization is the only criterion for SAE classification and the below listed criteria are met.

SAE's are not reported (but are documented as AE's if they meet the AE documentation criteria) if the only reason of SAE classification is:

o hospitalizations planned before entry into the clinical study
o elective treatment of a pre-existing condition
o routine treatment not associated with any deterioration in condition.

## 10.8 Expected adverse events

- Linked to disease under study: death, need for supportive renal dialysis, thrombosis, severe denutrition and severe infection

- Linked to the treatment by rituximab:

Expectedness will be determined according to the Summary of Product Characteristics for Truxima®.

The table below, extracted from the current SMPc of Truxima® describe the serious adverse drug reaction reported in clinical trials or during post-marketing surveillance in patients with non-Hodgkin's lymphoma (NHL) and chronic lymphoytic leukaemia (CLL) disease treated with rituximab monotherapy/maintenance or in combination with chemotherapy. Within each frequency grouping, undesirable effects are presented in order of decreasing seriousness. Frequencies are defined as very common (≥ 1/10), common (≥ 1/100 to < 1/10), uncommon (≥ 1/1,000 to < 1/100), rare (≥ 1/10,000 to < 1/1000), very rare (< 1/10,000) and not known (cannot be estimated from the available data).

| System organ class | Very common | Common | Uncommon | Rare | Very Rare | Not known |
|---|---|---|---|---|---|---|
| **Infections and infestations** | bacterial infection, viral infections, +bronchitis | sepsis, +pneumonia, +febrile infection, +herpes zoster, +respiratory tract infection, fungal infections, infections of unknown etiology, +acute bronchitis, +sinusitis, hepatitis B[1] | | serious viral infection[2] Pneumocystis jirovecii | PML | |
| **Blood and lymphatic system disorders** | neutropenia, leucopenia, +febrile neutropenia, +thrombocytopenia | anaemia, +pancytopenia, +granulocytopenia | coagulation disorders, aplastic anaemia, haemolytic anaemia, lymphadenopathy | | transient increase in serum IgM levels[3] | late neutropenia[3] |
| **Immune system disorders** | infusion related reactions[4], angioedema | hypersensitivity | | anaphylaxis | tumour lysis syndrome, cytokine release syndrome[4], serum sickness | infusion-related acute reversible thrombocytopenia[4] |

| System organ class | Very common | Common | Uncommon | Rare | Very Rare | Not known |
|---|---|---|---|---|---|---|
| **Metabolism and nutrition disorders** | | hyperglycaemia, weight decrease, peripheral oedema, face oedema, increased LDH, hypocalcaemia | | | | |
| **Psychiatric disorders** | | | depression, nervousness, | | | |
| **Nervous system disorders** | | paraesthesia, hypoaesthesia, agitation, insomnia, | dysgeusia | | peripheral neuropathy, facial nerve palsy[5] | cranial neuropathy, loss of other senses[5] |
| | | vasodilatation, dizziness, anxiety | | | | |
| **Eye disorders** | | lacrimation disorder, conjunctivitis | | | severe vision loss[5] | |
| **Ear and labyrinth disorders** | | tinnitus, ear pain | | | | hearing loss[5] |
| **Cardiac disorders** | | +myocardial infarction[4 and 6], arrhythmia, +atrial fibrillation, tachycardia, +cardiac disorder | +left ventricular failure, +supraventri-cular tachycardia, +ventricular tachycardia, +angina, +myocardial ischaemia, bradycardia | severe cardiac disoders[4 and 6] | heart failure[4 and 6] | |
| **Vascular disorders** | | hypertension, orthostatic hypotension, hypotension | | | vasculitis (predominat el y cutaneous), leukocytocla st ic vasculiti | |
| **Respiratory, thoracic and mediastinal disorders** | | bronchospasm[4], respiratory disease, chest pain, dyspnoea, increased cough, rhinitis | asthma, bronchiolitis obliterans, lung disorder, hypoxia | interstitial lung disease[7] | respiratory failure[4] | lung infiltration |
| **Gastrointestinal disorders** | nausea | vomiting , diarrhoea, abdominal pain, dysphagia, stomatitis, constipation, dyspepsia, anorexia, throat irritation | abdominal enlargement | | gastro-intesti n al perforation[7] | |

| System organ class | Very common | Common | Uncommon | Rare | Very Rare | Not known |
|---|---|---|---|---|---|---|
| **Skin and Subcutaneous tissue disorders** | pruritus, rash, $^{+}$alopecia | urticaria, sweating, night sweats, $^{+}$skin disorder | | | severe bullous skin reactions, Stevens-Johns on Syndrome toxic epidermal necrolysis (Lyell's Syndrome)[7], | |
| **Musculoskeletal, connective tissue and bone disorders** | | hypertonia, myalgia, arthralgia, back pain, neck pain, pain | | | | |
| **Renal and urinary disorders** | | | | | renal failure[4] | |
| **General** | fever , | tumour pain, | infusion site | | | |
| **disorders and administrationsite conditions** | chills, asthenia, headache | flushing, malaise, cold syndrome, $^{+}$fatigue, $^{+}$shivering, $^{+}$multi-organ failure[4] | pain | | | |
| **Investigations** | decreased IgG levels | | | | | |

For each term, the frequency count was based on reactions of all grades (from mild to severe), except for terms marked with "+" where the frequency count was based only on severe (≥ grade 3 NCI common toxicity criteria) reactions. Only the highest frequency observed in the trials is reported

[1] includes reactivation and primary infections; frequency based on R-FC regimen in relapsed/refractory CLL

[2] see also section infection in the SMPc

[3] see also section haematologic adverse reactions in the SMPC

[4] see also section infusion-related reactions in the SMPc. Rarely fatal cases reported

[5] signs and symptoms of cranial neuropathy. Occurred at various times up to several months after completion of rituximab therapy

[6] observed mainly in patients with prior cardiac condition and/or cardiotoxic chemotherapy and were mostly associated with infusion-related reactions

[7] includes fatal cases

The most frequent reported or observed serious adverse drug reactions were:

-IRRs (including cytokine-release syndrome, tumour-lysis syndrome)

-Infections

-Cardiovascular events

The overall safety profile of rituximab in rheumatoid arthritis is based on data from patients from clinical trials and from post-marketing surveillance.

The frequencies of ADRs reported with rituximab alone or in combination with chemotherapy are summarized in the table below, extracted from the current SMPc. Within each frequency grouping, undesirable effects are presented in order of decreasing seriousness. Frequencies are defined as very common (≥ 1/10), common (≥ 1/100 to < 1/10), uncommon (≥ 1/1,000 to < 1/100), rare (≥ 1/10,000 to < 1/1000), very rare (< 1/10,000) and not known (cannot be estimated from the available data).

The ADRs identified only during post-marketing surveillance, and for which a frequency could not be estimated, are listed under "not known".

| System organ class | Very common | Common | Uncommon | Rare | Very rare |
|---|---|---|---|---|---|
| **Infections and infestations** | upper respiratory tract infection, urinary tract infections | bronchitis, sinusitis, gastroenteritis, tineapedis | | | PML, reactivation of hepatitis B |
| **Blood and lymphatic system disorders** | | neutropenia[1] | | late neutropenia[2] | serum sickness-like reaction |
| **Immune system disorders**<br>**General disorders and administration site conditions** | [3]infusion related reactions (hypertension, nausea, rash, pyrexia, pruritus, urticaria, throat | | [3]infusion related reactions (generalised oedema, bronchospasm, wheezing, laryngeal | | |

| | | | | | |
|---|---|---|---|---|---|
| | irritation, hot flush, hypotension, rhinitis, rigors, tachycardia, fatigue, oropharyngeal pain, peripheral oedema, erythma) | | oedema, angioneurotic oedema, generalised pruritis, anaphylaxis, anaphylactoid reaction) | | |
| **Metabolism and nutritional Disorders** | | hypercholesterolemia | | | |
| **Psychiatric disorders** | | depression, anxiety | | | |
| **System organ class** | **Very common** | **Common** | **Uncommon** | **Rare** | **Very rare** |
| **Nervous system disorders** | headache | paraesthesia, migraine, dizziness, sciatica | | | |
| **Cardiac disorders** | | | | angina pectoris, atrial fibrillation, heart failure, myocardial infarction | atrial flutter |
| **Gastrointestinal disorders** | | dyspepsia, diarrhoea, gastro-oesophageal reflux, mouth ulceration, upper abdominal pain | | | |
| **Skin and subcutaneous tissue disorders** | | alopecia | | | toxic epidermal necrolysis (Lyell's Syndrome), Stevens-Johnson Syndrome[5] |
| **Musculo-skeletal disorders** | | arthralgia / musculoskeletal pain, osteoarthritis, bursitis | | | |
| **Investigations** | decreased IgM levels[4] | decreased IgG levels[4] | | | |

[1] Frequency category derived from laboratory values collected as part of routine laboratory monitoring in clinical trials

[2] Frequency category derived from post-marketing data.

[3] Reactions occurring during or within 24 hours of infusion. See also infusion-related reactions below. IRRs may occur as a result of hypersensitivity and/or to the mechanism of action.

[4] Includes observations collected as part of routine laboratory monitoring.

[5] Includes fatal cases

- Linked to the antihypertensive treatments (angiotensin-converting enzyme inhibitor (ACEI)): cough, elevated blood potassium levels, low blood pressure, dizziness, headache, drowsiness, weakness, abnormal taste (metallic or salty taste), rash, chest pain, increased uric acid levels, sun sensitivity, increased BUN and creatinine levels, kidney failure, allergic

reactions, pancreatitis, liver dysfunction, decrease in white blood cells, swelling of tissues (angioedema).

- Linked to the antihypertensive treatments (angiotensin 2 receptor antagonist): cough, elevated potassium levels in the blood (hyperkalemia), low blood pressure, dizziness, headache, drowsiness, diarrhea, abnormal taste sensation (metallic or salty taste), rash, orthostatic hypotension (low blood pressure upon standing), fatigue, indigestion and upper respiratory tract infection.

- Linked to the diuretics: the most common are hyponatremia and dehydration, hypovolemia and functional renal failure (decrease glomerular filtration), orthostatic hypotension, dizziness and weakness in the elderly, hyperuricemia.

- Linked to the statins: the most common are digestive troubles and myopathy with myalgia.

- Linked to the blood samples: pain, local infection, hematomas

- Linked to the vaccination (pneumococcal and influenza vaccine): Expectedness will be determined according to the Summary of Product Characteristics for Pneumovax and Influvac (influenza vaccine)
- Linked to TAP scanner: Bleeding, soreness or swelling may develop at injection site. Allergic reactions to the radiopharmaceutical.
- Linked to the concomitant medication if applicable (perfalgan 1g IV, Polaramine 5 mg and Solumedrol 100 mg): Expectedness will be determined according to the respective Summary of Product Characteristics.

### 10.9 Annual reporting by the sponsor to the Agency

1.Regarding investigational medicinal products other than placebo, the sponsor shall submit annually through the database referred to in Article 10.5.1 to the Agency a report on the safety of each investigational medicinal product used in a clinical trial for which it is the sponsor.

2.In the case of a clinical trial involving the use of more than one investigational medicinal product, the sponsor may, if provided for in the protocol, submit a single safety report on all investigational medicinal products used in that clinical trial.

3. The annual report shall only contain aggregate and anonymised data.

4. The obligation referred to in paragraph 1 starts with the first authorisation of a clinical trial in accordance with this Regulation. It ends with the end of the last clinical trial conducted by the sponsor with the investigational medicinal product.

### 10.10 Withdrawal of Study Participants

The researchers may decide to withdraw the patient from the study if:

- The medical problem becomes worse.
- The researchers believe that participation in the research is no longer safe for the patient.
- The researchers believe that other treatment may be more helpful.
- The sponsor or the EMA stops the research for the safety of the participants.
- The sponsor cancels the research.
- The patient is unable to keep appointments or to follow the researcher’s instructions.

### 10.11 Pregnancy

If a woman becomes pregnant during the course of this study, the patient will be excluded.

All medical decisions concerning her and her fetus will be in Multidisciplinary consultation meeting and will be presented thereafter.

- Reporting of pregnancy

Irrespective of the treatment received by the subject, any pregnancy occurring during the study must be reported immediately.

Pregnancies must be reported on the Sponsor Pregnancy form, which is send to Sponsor pharmacovigilance (see contact details provided on the pregnancy form), and on the AE page in the e-CRF.

- Follow-up of pregnancy

Any pregnancy must be followed to its conclusion and its outcome must be reported to the Sponsor pharmacovigilance.

### 10.12 Data safety monitoring board (DSMB)

As Truxima is an approved EMEA and FDA treatment, we do not anticipate any impediments to patient enrollment or significant adverse or serious adverse events. As a result, no data safety advisory board is required during the study.

## 11. Data collection

### 11.1 Study records

A case report form (CRF) will be specifically designed for study data collection. The Clinical Research Assistant responsible for the sponsorship of the study, and the Data Manager of the Department of Clinical Research and Innovation at Nice will design the CRF, in coordination with, and under the responsibility of, the principal investigator.

### 11.2 Database

The study data will be recorded in the electronic record (e-CRF). This will be implemented by the Data Manager of the Department of Clinical Research and Innovation using RedCap® software from the finalized paper CRF. Parameter specification and the implementation of the e-CRF for data collection, including users training, will be the responsibility of the Department of Clinical Research and Innovation.

The investigators, together with the clinical research assistants, in each center will take responsibility for data collection and entering it directly into the e-CRF. The data will be securely stored, with specific access rights granted to members of the study team according to their role.

### 11.3 Data quality control

Data quality control will be performed on the e-CRF, using the patient medical file, by the sponsor during the planned monitoring visits by the Department of Clinical Research and Innovation's Clinical Research Officer.

Once the final data have been entered, checks for their validity and coherence will be performed by the Data Manager of the Department of Clinical Research and Innovation and requests for

verification issued. Throughout the study any modifications to the database will be recorded, enabling a full audit trail.
At the end of the quality control process, the database will be frozen and signed off by the principal investigator, the data manager and the head of biometric department at the Department of Clinical Research and Innovation. No modification of the data will be possible after this time.

The frozen database, together with the data management report, will then be transferred to the statistician for analysis.

## 12. Statistical analysis

### 12.1 Analysis strategy

The statistical analyses will be performed by the biostatistician of the Delegation of Clinical Research and Innovation (DRCI) at the Nice center.
Before each analysis is performed, the conditions for the application of the tests that were used will be verified. The various tests will be considered significant at a threshold of 5% (unless otherwise specified). Continuous variables will be described using the number of observations (N), arithmetic mean (Mean), standard deviation, minimum (MIN), median (Median), and maximum (MAX) values. Categorical variables will be summarized by absolute (N) and relative frequencies (%). The statistical analysis will be performed using SAS Enterprise Guide 5.1 software (Copyright (c) 2012 by SAS Institute Inc., Cary, NC, USA). The statistical analysis will be performed using SAS Enterprise Guide 5.1 software (Copyright (c) 2012 by SAS Institute Inc., Cary, NC, USA).
Population analyzed:
The study patients will be analyzed according to the intention to treat principle (ITT). Each patient will be analyzed as a part of the group to which he or she was assigned at randomization.
A *per protocol* analysis will also be performed, though the results of this analysis cannot be substituted for those of the intention to treat analysis.

Missing data:
The “last observation carried forward” (LOCF) strategy will be used to impute the missing data for the principal objective and, when possible, for the secondary objectives.

Disposition of patients:

According to the CONSORT 2010 statements, a flow diagram of the progress through the phases (enrollment, allocation, follow-up, and analysis) of this study will be presented. The number of screening failures and reasons for screening failures will be summarized. The characteristics of patients lost to follow-up over the course of the study will be reported.

## 12.2 Descriptive analysis

The statistical analysis will first present a descriptive analysis of the study population and their measured parameters with absolute and relative frequencies (and their 95% confidence intervals) for the categorical variables, and evaluation of means and distributions, medians and inter quartiles for the quantitative variables.
As the CONSORT guidelines recommend, the principal characteristics of the patients will be compared between the two groups at inclusion, but no statistical analysis of this will be performed. The comparability of the two arms will be assessed clinically rather than statistically. A flow chart showing the number of eligible patients, the number of patients included, and the number of patients randomized will be presented. The characteristics of patients lost to follow up over the course of the study will be described.

## 12.3 Outcomes analyses

### 12.3.1 Analysis of the primary objective:

The primary objective of this study is to compare the clinical remission rate over one year between patients with and without personalized treatment.
The clinical remission rate at M12 will be compared between the groups using a Chi-square test or Fisher's exact test in case of small sample.
If necessary, a multivariate logistic regression analysis will be performed to assess the relationship between the clinical remission and the treatment group, adjusted for the potential confounding factors.

### 12.3.2 Analysis of the main secondary objective:

The secondary objectives consist in comparing several parameters between both treatment groups (GEMRITUX versus Personalized treatment) at different study timepoints according to each specific objective. The secondary assessment criteria can be grouped into two categories:

- Objectives 1, 2 and 3: binary criteria (remission clinical or immunological), compared at different timepoints between the two groups. Comparisons will be performed using a Chi-square test or Fisher’s exact test in case of small sample.
- Objectives 4, 6 and 8: quantitative criteria compared at different timepoints between the two groups. Comparisons will be performed using a student t-test (or mann-whitney rank sum test in case of non-parametric variables).

- Objectives 5, 7: Quantitative criteria evolution (changes from baseline), defined as the as the difference between the $M_x$ and $D_0$ values, Δvariable = Variable $M_x$ – Variable $D_0$. Analyses will use an analysis of covariance which takes into account the initial values as a covariate. The dependent variable will be the Δvariable and the variable of interest the treatment group.

- Objective 9: Severe infections will be described by type and their frequencies, absolute and relative, presented. The rate of patients presenting with at least one severe infection during follow-up will be compare between the two groups using a Chi-square test or Fisher’s exact test in case of small sample.

Further multivariate analyses will be eventually discussed in case of imbalanced potential confounding factors between the groups.

## 13. Feasibility

This protocol is supported by the *Centre de Référence Maladie rare “Syndrome néphrotique”* (see annexe).

The centers participating in this network follow about 2 new MN patients each year. In order to achieve the objective of recruiting 64 patients in four years, 22 centers will have to actively recruit on average one patient each year.

Moreover, the experience from the PHRC GEMRITUX (NCT01508468) and the PHRC PRAM-KT (PHRC2011-A01302-39, NCT01897961) showed the ability of our centers to recruit such patients. We are currently conducting an observational study (SOURIS 2014-A00886-41) involving 5 centers that recruited 33 patients in 2 years with the same inclusion criteria.

## 14. Independent data-monitoring committee

The independent data monitoring committee is a consultative committee charged with advising the study sponsor on the benefit/risk ratio of the conduct of a clinical trial.

Its members, all competent in the conduct of clinical trials (pathology, methodology...) are not directly involved in the study.

The selection of its membership is made by the sponsor in collaboration with the coordinating investigator.

They are nominated by the sponsor and their term of office corresponds to the length of the study.

They undertake their duties voluntarily, and agree to respect the confidentiality of the study data.

The committee will receive all versions of the protocol, the annual safety report, and may be consulted at any time by the sponsor if a suspected unexpected severe adverse reaction or unwanted effect is particularly difficult to analyze, and/or if the data may change the apparent risk/benefit ratio during the course of the study.

The committee will analyze the data sent to it, and may demand additional information. It then makes a recommendation about the future of the study (continue, modify, halt etc).

The committee will meet annually.

Each associate investigator declaring one of the following events:

- commencement of supportive renal dialysis,
- or a non-fatal cardiovascular event: myocardial infarct, cerebrovascular accident, coronary or peripheral revascularization procedure, amputation, hospitalization for heart failure, cardiac arrest and resuscitation),
- or cardiovascular mortality,
- or non-cardiovascular mortality.

will complete a specific form and supply a hospital summary or consultation record that documents the event. These documents will be sent to the Nice DRCI, who will pass them to the independent data monitoring committee before each of its annual meetings.

If there is evidence that brings the safety of the study intervention into question is identified, the protocol may be suspended.

## 15. Study resources

**Biology**

The greatest part of the requested budget is devoted to develop new tests: epitope profile characterization, B and T cells count with TReg and Memory B cells, immunomonitoring of rituximab, cytokine profile determination. To these purposes, a 50% of lab technician will be employed.

**Study coordination and the analyses of the results**

A 50% of Study Coordinator will be employed at the coordinating center for the duration of the study. His tasks will be to coordinate the study, collect and enter data onsite.

In all investigators' centers, one vacation per month clinical research assistant for each visit of patient will be dedicated for data collection and data entry.

A budget is dedicated:

- for the creation of the database, the data management and the statistical analyses performed by a data manager and a statistician,
- for the monitoring by a monitor,
- for the ethical and logistical follow-up by a project manager,
- for the data safety by a pharmacist.

For the data committee, a budget will be allocated to the 4 members for their participation (one committee by year).

**Other costs**

- Travel expenses for the study monitoring by the monitor
- Samples shipping
- Lab supplies
- Conference fees and publication expenses (translation)
- Insurance
- Paper work

## 16. Ethical and regulatory requirements:

### 16.1 Regulation

All investigators involved in the project lead the trial in full compliance with:

- <u>The Helsinki declaration:</u>

The Declaration of Helsinki was established by the World Medical Association in June 1964 (recommendations to guide physicians in biomedical research), revised in Tokyo in October 1975 in

Venice in October 1983 in Hong Kong in September 1989, Somerset West in October 1996 and in Edinburgh in October 2000.

- The European Directive on Good Clinical Practice in the Conduct of Clinical Trials:

Directive 2001/20/EC was adopted 4 April 2001 by the European Parliament and the Council of the European Union, and requires member states to approximate their laws, regulations and administrative provisions relating to the implementation of good clinical practice in the conduct of clinical trials on medicinal products for human use.

In France, drug testing is governed by practice guidelines laid out by the health minister and by decisions of the national agency for medicines and health products (Agence National de Sécurité du Médicaments et produits de santé, the "ANSM") for products referred to in Article Ls 5311 -1 (medical devices, blood products ...).

Recommendations for good clinical practice apply: see ICH Topic E6, CPMP/ICH/135/95.

- Legislative and regulatory:

Public Health Law 2004-806 of August 9, 2004 (Articles L.1121-1 to L.1126-7). This Act amends the so-called Huriet-Sérusclat law 88-1138 of 20 December 1988, governing biomedical research performed for the development of biological and medical knowledge. The Enforcement Decree of the Act was published April 26, 2006 under number 2006-477. The scope of the law has been redefined, the Public Health Code (CSP) applies to research "organized and practiced on the human being for the development of biological and medical knowledge."

Decree n ° 2007-1220 of 10 August 2007 on the collection and preservation of the human body parts for scientific ends, amending the Code of Public Health (Regulations) Code of public health, in particular Articles L. 1232-6 and L. 1243-9.

## 16.2 Ethical considerations:

- Patient information and consent:

Prior to the beginning of the research, the investigator is responsible for informing the person on the conduct of the trial and its consequences. The patient should also be informed of any medical alternatives, the terms of medical care provided at the end of research as well as his right to be informed on the overall results of the research.

Consent, freely and express will be collected in writing (article L-1122-1 of the Code of Public Health) knowing that the patient will have the opportunity to ask questions and be informed of his right to

refuse to participate in research and withdraw at any time of the test without giving any justification and without prejudice thereby.

The investigator will sign the informed consent, in duplicate, retaining the original, and giving a copy to the patient.

- Medical examination and affiliation to a social security system:

Medical examination and affiliation to a social security system of a person who may participate in a trial is required for all biomedical research (Article L1121-11 of the Code of Public Health).

- Data confidentiality:

Medical and non-medical staffs involved in the trial are subject to medical and professional confidentiality with the data collected during the study patient.

Documents relating to the trial should be stored in a cupboard or lockable room.

### 16.3 Registration of the trial in the database reference:

The trial was registered in the national database managed by the National Security Agency for Medicines and Health Products (ANSM), which lists all biomedical research by French developers.

### 16.4 Role of the sponsor in the implementation of the project:

- Liability Insurance:

The sponsor has insurance liability to the Hospital Mutual Insurance Company (SHAM) for the realization of this trial.

- Request authorization from the Comité de Protection des Personnes (CPP) and ANSM:

This study received the 07/05/2019 the approval of the CPP SOOM II and 28/09/2018 the authorization of the ANSM.

After the commencement of the research, any substantial modification thereof to the initiative of the sponsor must obtain, prior to its implementation, a favorable opinion of the CPP and authorization of ANSM. (Article L1123-9).

Statement to the National Commission for Computing and Liberties:

The processing of this data is in accordance with the Data Protection Act n ° 78-17 of January 6, 1978 as amended and as well as with Regulation (EU) 2016/679 of the European Parliament and of the Council of April 27, 2016 applicable from May 25 2018 (General Regulation on Data Protection,

RGPD). This treatment is declared in the register of treatment activities of the University Hospital of Nice.

By the deliberation n ° 2018-153 of May 3rd, 2018 relating to the homologation of a reference methodology for the processing of personal data operated within the framework of the researches in the field of the health requiring the collection of express or written consent of the person concerned (MR-001), the CNIL research statement will consist of a commitment to this reference methodology.

Data collected before the opposition of a patient in the study may not be erased and may continue to be treated under the conditions provided by the research (Articles 17.3.c and 17.3.d of the RGPD).

The details of the Delegate for the Protection of Persons are as follows:

- email: dpo@chu-nice.fr
- address: Mail to the attention of the Data Protection Officer (DPO), Cimiez Hospital, 4 avenue Reine Victoria - CS 91179 - 06003 Nice Cedex 1.

### 16.5 Projects and results

- Trial beginning:

The beginning of the study is the first patient included in the trial.

The first inclusion in the study should be immediately communicated to the ANSM and the CPP.

Research must be started within one year after the notification of the CPP which case the notice will lapse.

- Monitoring and inspection:

Quality control will be done by a Clinical Research Associate at the Direction de la Recherche Clinique et de l'Innovation appointed by the sponsor.

The nature and frequency of monitoring will be based on the procedure for setting the level of monitoring risk-based established and depend on the number of patients included, the rate of inclusions and difficulties encountered during the realization of the study.

CRA of the DRCI should verify with the investigator:

- Checking the accuracy and completeness of the CRF entries, source documents and other trial-related records against each other.
- Verifying that written informed consent was obtained before each subject's participation in the trial.
- SAE

For that point, the investigator agrees to make available for the CRA monitoring:

 The medical records of patients

 The Case Report Form

 The consent forms of included patients

The purposes of trial monitoring are to verify that:

(a) The rights and well-being of human subjects are protected.

(b) The reported trial data are accurate, complete, and verifiable from source documents.

(c) The conduct of the trial is in compliance with the currently approved protocol/amendment(s), with GCP, and with the applicable regulatory requirement(s).

An inspection is a formal control by the MSNA in order to evaluate the admissibility of clinical data, verify compliance with legislation and the absence of fraud. Inspectors check documents, logistics, records and any other resource that the authorities consider associated with the clinical trial and that may be on the scene of the trial, at the sponsor and / or local the provider service agency (Contract Research Organizations CRO) or other establishments if necessary.

- Final Scientific Report and end of study:

At the end of the test, it is imperative that the sponsor shall notify the CPP and ANSM the end of the research (date of last visit of the last patient) within 90 days.

The principal investigator shall prepare the final scientific report of the project which must contain the results and clinical implications of the study. The investigator will be invited to present these results to the Scientific Committee of the CHU of Nice.

A summary of the final report shall be communicated to the ANSM in the year following the end of the research.

Furthermore, at the end of the trial, the sponsor should informed associated centers and the CPP. According to the Law of August 9, 2004, patients may ask the investigator summarizes the overall results of the research as described in the disclosure documents.

- Publication :

Any communication or publication must mention the sponsor of the study that is to say, the CHU of Nice and the funding source (PHRC). In this respect, the investigator must submit a copy of these communications and / or publications to the Department of Research and Innovation of Nice.

- Storage :

Accordance with Good Clinical Practice, at the end of the trial, all documents relating to the Protocol shall be archived for a period of 15 years by the principal investigator.

All these documents must be located in a lockable room providing adequate safeguards against fire, water damage, light, or malice.

## 17. Rules governing publication

In accordance with the article “Sponsorship, authorship and accountability, N Engl J Med 2001 ;345 :825-7”, the PMMN steering committee (SC) participated in the design of the protocol, owns the database, and is free to interpret and publish the data.

Abstracts or publications using PMMN study data will be submitted with the prior agreement of the steering committee president. These abstracts and publications will be prepared by writing groups appointed by the steering committee. Every publication must be reviewed by the steering committee whose approval must be obtained before submission. The study coordinator will be the first author of the principal study; the last author will be the steering committee president. The coordinator of the endocrinologists and nutritionists may choose to be joint-first or joint-last author. Any PMMN study co-investigator may propose an ancillary study to the steering committee, addressing this request in writing to the steering committee president. These proposals will be reviewed for methodological soundness by the study's biostatistical unit before being evaluated by the steering committee. Specific additional funding must be obtained to finance these studies. Any data necessary for the ancillary study will then be extracted from the database and sent to the responsible investigator. No ancillary study is to be published before publication of the principal study. The order of the list of authorship will be defined by the steering committee in agreement with the responsible investigator of the ancillary study. The responsible investigator for the ancillary study will sign the paper as first author, and the steering committee president will be last author.

The PMMN study defines a primary objective, secondary objectives, a methodology, and a statistical schema. All of the co-investigators participated in this research leading to its results and conclusions. These conclusions are the result of the efforts of the whole group, and are not simply a juxtaposition of myriad results obtained by many researchers participating in the study. For this reason, the investigators are not at liberty to publish the isolated results from their own institution. The database is the property of the entire group, under the coordination of the steering committee president. If an investigator wishes to propose an ancillary study, whether or not it uses biological specimens, it must be in conformance with this principle.

A copy of the publication will be submitted to Nice CHU, the study sponsor, who will be necessarily be cited. Apart from the coordinating investigators, the list of authorship will be determined pro rata according to the number of patients recruited and analyzed in the publication in question: two co-authors per center having recruited at least 4 patients, and one co-author for each center recruiting between 2 and 4 patients.

The protocol will be registered on the American clinical trials register clinicaltrials.gov

## 18. References

1. Lassalle M, Ayav C, Frimat L, Jacquelinet C, Couchoud C, Au Nom du Registre R. The essential of 2012 results from the French Renal Epidemiology and Information Network (REIN) ESRD registry. Nephrologie & therapeutique. 2015 Apr;11(2):78-87. PubMed PMID: 25457107.

2. Simon N, Courouce AM, Lemarrec N, Trepo C, Ducamp S. A twelve year natural history of hepatitis C virus infection in hemodialyzed patients. Kidney international. 1994 Aug;46(2):504-11. PubMed PMID: 7967364.

3. Simon P, Ramee MP, Boulahrouz R, Stanescu C, Charasse C, Ang KS, et al. Epidemiologic data of primary glomerular diseases in western France. Kidney international. 2004 Sep;66(3):905-8. PubMed PMID: 15327379.

4. Ponticelli C. Membranous nephropathy. Journal of nephrology. 2007 May-Jun;20(3):268-87. PubMed PMID: 17557260.

5. Glassock RJ. The pathogenesis of idiopathic membranous nephropathy: a 50-year odyssey. American journal of kidney diseases : the official journal of the National Kidney Foundation. 2010 Jul;56(1):157-67. PubMed PMID: 20378220.

6. Debiec H, Guigonis V, Mougenot B, Decobert F, Haymann JP, Bensman A, et al. Antenatal membranous glomerulonephritis due to anti-neutral endopeptidase antibodies. The New England journal of medicine. 2002 Jun 27;346(26):2053-60. PubMed PMID: 12087141.

7. Beck LH, Jr., Bonegio RG, Lambeau G, Beck DM, Powell DW, Cummins TD, et al. M-type phospholipase A2 receptor as target antigen in idiopathic membranous nephropathy. The New England journal of medicine. 2009 Jul 02;361(1):11-21. PubMed PMID: 19571279. Pubmed Central PMCID: 2762083.

8. Tomas NM, Beck LH, Jr., Meyer-Schwesinger C, Seitz-Polski B, Ma H, Zahner G, et al. Thrombospondin type-1 domain-containing 7A in idiopathic membranous nephropathy. The New

England journal of medicine. 2014 Dec 11;371(24):2277-87. PubMed PMID: 25394321. Pubmed Central PMCID: 4278759.

9. Beck LH, Jr., Fervenza FC, Beck DM, Bonegio RG, Malik FA, Erickson SB, et al. Rituximab-induced depletion of anti-PLA2R autoantibodies predicts response in membranous nephropathy. Journal of the American Society of Nephrology : JASN. 2011 Aug;22(8):1543-50. PubMed PMID: 21784898. Pubmed Central PMCID: 3148709.

10. Ronco P, Debiec H. Pathophysiological advances in membranous nephropathy: time for a shift in patient's care. Lancet. 2015 May 16;385(9981):1983-92. PubMed PMID: 26090644.

11. Schieppati A, Mosconi L, Perna A, Mecca G, Bertani T, Garattini S, et al. Prognosis of untreated patients with idiopathic membranous nephropathy. The New England journal of medicine. 1993 Jul 08;329(2):85-9. PubMed PMID: 8510707.

12. Glassock RJ. Diagnosis and natural course of membranous nephropathy. Seminars in nephrology. 2003 Jul;23(4):324-32. PubMed PMID: 12923720.

13. Kanigicherla D, Gummadova J, McKenzie EA, Roberts SA, Harris S, Nikam M, et al. Anti-PLA2R antibodies measured by ELISA predict long-term outcome in a prevalent population of patients with idiopathic membranous nephropathy. Kidney international. 2013 May;83(5):940-8. PubMed PMID: 23364522.

14. Hoxha E, Thiele I, Zahner G, Panzer U, Harendza S, Stahl RA. Phospholipase A2 receptor autoantibodies and clinical outcome in patients with primary membranous nephropathy. Journal of the American Society of Nephrology : JASN. 2014 Jun;25(6):1357-66. PubMed PMID: 24610926. Pubmed Central PMCID: 4033365.

15. Radhakrishnan J, Cattran DC. The KDIGO practice guideline on glomerulonephritis: reading between the (guide)lines--application to the individual patient. Kidney international. 2012 Oct;82(8):840-56. PubMed PMID: 22895519.

16. Remuzzi G, Chiurchiu C, Abbate M, Brusegan V, Bontempelli M, Ruggenenti P. Rituximab for idiopathic membranous nephropathy. Lancet. 2002 Sep 21;360(9337):923-4. PubMed PMID: 12354476.

17. Fervenza FC, Cosio FG, Erickson SB, Specks U, Herzenberg AM, Dillon JJ, et al. Rituximab treatment of idiopathic membranous nephropathy. Kidney international. 2008 Jan;73(1):117-25. PubMed PMID: 17943078.

18. Dahan K, Debiec H, Plaisier E, Cachanado M, Rousseau A, Wakselman L, et al. Rituximab for Severe Membranous Nephropathy: A 6-Month Trial with Extended Follow-Up. Journal of the American Society of Nephrology : JASN. 2016 Jun 27. PubMed PMID: 27352623.

19. Seitz-Polski B, Payre C, Ambrosetti D, Albano L, Cassuto-Viguier E, Berguignat M, et al. Prediction of membranous nephropathy recurrence after transplantation by monitoring of anti-PLA2R1 (M-type phospholipase A2 receptor) autoantibodies: a case series of 15 patients. Nephrology, dialysis, transplantation : official publication of the European Dialysis and Transplant Association - European Renal Association. 2014 Jul 25. PubMed PMID: 25063424.

20. Seitz-Polski B, Dolla G, Payre C, Tomas NM, Lochouarn M, Jeammet L, et al. Cross-reactivity of anti-PLA2R1 autoantibodies to rabbit and mouse PLA2R1 antigens and development of two novel ELISAs with different diagnostic performances in idiopathic membranous nephropathy. Biochimie. 2015 Nov;118:104-15. PubMed PMID: 26296473.

21. Ancian P, Lambeau G, Mattei MG, Lazdunski M. The human 180-kDa receptor for secretory phospholipases A2. Molecular cloning, identification of a secreted soluble form, expression, and chromosomal localization. The Journal of biological chemistry. 1995 Apr 14;270(15):8963-70. PubMed PMID: 7721806.

22. Fresquet M, Jowitt TA, Gummadova J, Collins R, O'Cualain R, McKenzie EA, et al. Identification of a major epitope recognized by PLA2R autoantibodies in primary membranous nephropathy. Journal of the American Society of Nephrology : JASN. 2015 Feb;26(2):302-13. PubMed PMID: 25288605. Pubmed Central PMCID: 4310666.

23. Kao L, Lam V, Waldman M, Glassock RJ, Zhu Q. Identification of the Immunodominant Epitope Region in Phospholipase A2 Receptor-Mediating Autoantibody Binding in Idiopathic Membranous Nephropathy. Journal of the American Society of Nephrology : JASN. 2014 Sep 9. PubMed PMID: 25205735.

24. Seitz-Polski B, Dolla G, Payre C, Girard CA, Polidori J, Zorzi K, et al. Epitope Spreading of Autoantibody Response to PLA2R Associates with Poor Prognosis in Membranous Nephropathy. Journal of the American Society of Nephrology : JASN. 2016 May;27(5):1517-33. PubMed PMID: 26567246. Pubmed Central PMCID: 4849812.

25. Cattran D, Brenchley P. Membranous nephropathy: thinking through the therapeutic options. Nephrology, dialysis, transplantation : official publication of the European Dialysis and Transplant Association - European Renal Association. 2017 Jan 01;32(suppl_1):i22-i9. PubMed PMID: 28391348.

26. Cravedi P, Ruggenenti P, Sghirlanzoni MC, Remuzzi G. Titrating rituximab to circulating B cells to optimize lymphocytolytic therapy in idiopathic membranous nephropathy. Clinical journal of the American Society of Nephrology : CJASN. 2007 Sep;2(5):932-7. PubMed PMID: 17702725.

27. Fervenza FC, Abraham RS, Erickson SB, Irazabal MV, Eirin A, Specks U, et al. Rituximab therapy in idiopathic membranous nephropathy: a 2-year study. Clinical journal of the American Society of Nephrology : CJASN. 2010 Dec;5(12):2188-98. PubMed PMID: 20705965. Pubmed Central PMCID: 2994079.

28. Piro LD, White CA, Grillo-Lopez AJ, Janakiraman N, Saven A, Beck TM, et al. Extended Rituximab (anti-CD20 monoclonal antibody) therapy for relapsed or refractory low-grade or follicular non-Hodgkin's lymphoma. Annals of oncology : official journal of the European Society for Medical Oncology. 1999 Jun;10(6):655-61. PubMed PMID: 10442187.

29. Cartron G, Blasco H, Paintaud G, Watier H, Le Guellec C. Pharmacokinetics of rituximab and its clinical use: thought for the best use? Critical reviews in oncology/hematology. 2007 Apr;62(1):43-52. PubMed PMID: 17287129.

30. Cartron G, Trappe RU, Solal-Celigny P, Hallek M. Interindividual variability of response to rituximab: from biological origins to individualized therapies. Clinical cancer research : an official journal of the American Association for Cancer Research. 2011 Jan 01;17(1):19-30. PubMed PMID: 21208903.

31. Thurlings RM, Teng O, Vos K, Gerlag DM, Aarden L, Stapel SO, et al. Clinical response, pharmacokinetics, development of human anti-chimaeric antibodies, and synovial tissue response to rituximab treatment in patients with rheumatoid arthritis. Annals of the rheumatic diseases. 2010 Feb;69(2):409-12. PubMed PMID: 19596693.

32. Ruggenenti P, Debiec H, Ruggiero B, Chianca A, Pelle T, Gaspari F, et al. Anti-Phospholipase A2 Receptor Antibody Titer Predicts Post-Rituximab Outcome of Membranous Nephropathy. Journal of the American Society of Nephrology : JASN. 2015 Oct;26(10):2545-58. PubMed PMID: 25804280. Pubmed Central PMCID: 4587688.

33. Roccatello D, Sciascia S, Di Simone D, Solfietti L, Naretto C, Fenoglio R, et al. New insights into immune mechanisms underlying response to Rituximab in patients with membranous nephropathy: A prospective study and a review of the literature. Autoimmunity reviews. 2016 Jun;15(6):529-38. PubMed PMID: 26876383.

34. Rosenzwajg M, Languille E, Debiec H, Hygino J, Dahan K, Simon T, et al. B- and T-cell subpopulations in patients with severe idiopathic membranous nephropathy may predict an early response to rituximab. Kidney international. 2017 Jul;92(1):227-37. PubMed PMID: 28318628.

35. Collison LW, Workman CJ, Kuo TT, Boyd K, Wang Y, Vignali KM, et al. The inhibitory cytokine IL-35 contributes to regulatory T-cell function. Nature. 2007 Nov 22;450(7169):566-9. PubMed PMID: 18033300.

36. Colucci M, Carsetti R, Cascioli S, Casiraghi F, Perna A, Rava L, et al. B Cell Reconstitution after Rituximab Treatment in Idiopathic Nephrotic Syndrome. Journal of the American Society of Nephrology : JASN. 2016 Jun;27(6):1811-22. PubMed PMID: 26567244. Pubmed Central PMCID: 4884116.

Annex 1: All treatments uses in this study in usual practice will be in accordance with the last SmPC downloadable to internet website: http://base-donnees-publique.medicaments.gouv.fr
